# Metabolomic Signatures of Prediabetes in Mexican Americans: The Role of Genetics and Macronutrients

**DOI:** 10.1002/ggn2.202500032

**Published:** 2025-12-16

**Authors:** Shinhye Chung, Charles Evans, Charles F. Burant, Han Chen, Bing Yu, David Aguilar, Eric L. Brown, Craig L. Hanis, Goo Jun

**Affiliations:** ^1^ Human Genetics Center, Department of Epidemiology, School of Public Health The University of Texas Health Science Center at Houston Houston TX USA; ^2^ Michigan Regional Comprehensive Metabolomics Resource Core University of Michigan Ann Arbor MI USA; ^3^ Division of Cardiovascular Medicine University of Texas Southwestern Medical Center Dallas TX USA; ^4^ Center for Infectious Disease University of Texas Health Science Center at Houston Houston TX USA

**Keywords:** genetic association, gene‐environmental association, metabolite, mexican americans, nutrients, prediabetes

## Abstract

Prediabetes is one of the main health concerns in public health, and various etiological factors contribute to its onset. This study aimed to evaluate genetic associations and gene‐macronutrient interaction with prediabetes‐related metabolites to understand how genetic variation and dietary intake contribute to dysglycemia. We analyzed a total of 482 self‐identified Mexican American participants recruited from Starr County, Texas in 2018‐2019. Untargeted metabolomic profiling was performed using LC‐MS. Nutrient densities of six macronutrients were derived from a 106‐item food frequency questionnaire. Genetic associations for each metabolite were tested using Generalized linear Mixed Model Association Tests (GMMAT). Gene‐macronutrient interactions on prediabetes‐associated metabolites were assessed with the Mixed Model Association Test for GEne‐Environment Interaction (MAGEE). Age, gender, and BMI were included as covariates in all association tests. Among 308 named and 2,471 unnamed metabolites, 17 novel variant‐metabolite pairs were discovered, including rs10947898 in *LRFN2* associated with diacylglycerol DG32:1(p‐value: 8.95E‐09). Among 145 named and 687 unnamed metabolites after filtering, gene‐macronutrient interaction analyses identified seven named metabolites, including a variant(rs111251222) in *MXD3* that interacted with monounsaturated fat to influence eicosadienoic acid levels (Interaction p‐value: 9.88E‐09). Prediabetes and nutrient‐related metabolites in Mexican Americans showed significant genetic associations and gene‐nutrient interactions.

## Graphical abstract

We studied how genes and diet influence metabolites in Mexican Americans. We found new genetic links to 17 blood metabolites and discovered 7 interactions between genetic variants and macronutrients like protein and various types of fat. These findings could help personalize strategies for preventing diabetes based on genetic and dietary factors in Mexican Americans.

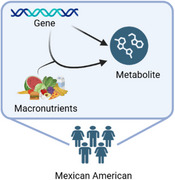

## 1 Introduction

Prediabetes, first conceptualized in the 1970s, is a reversible disorder that represents an intermediate state between normal glycemia and diabetes [1]. It is typically characterized as impaired glucose tolerance (IGT), impaired fasting glucose (IFG), or by increased hemoglobin A1c (HbA1c) levels, since dysglycemia can be diagnosed with three distinct glycemic measurements. Globally, approximately 12.0% of adults worldwide have IGT and 9.2% have IFG, and the prevalence of prediabetes continues to rise [2]. Although it is the first step of worsening glycemia, lifestyle modifications, including a healthy diet, can restore normoglycemia in individuals with prediabetes. Thus, identifying factors that contribute to the development of prediabetes could be essential to its prevention.

Genetic predisposition also plays a key role in the development of dysglycemia. The heritability of type 2 diabetes mellitus (T2D) is estimated to range from 30% to 70%, with approximately 20% of the heritability attributed to common variants [3]. Population‐based analyses of single‐nucleotide polymorphisms (SNPs) have identified over 700 loci associated with susceptibility to type 2 diabetes, including variants in *TCF7L2* located on chromosome 10 [3, 4, 5, 6, 7]. However, relatively few studies have focused on the genetic underpinnings of prediabetes. Individuals with fully developed diabetes often have all three glucose measures (fasting, 2‐hr, and HbA1c) exceeding the normal range, making it difficult to study different genetic pathologies contributing to the development of diabetes. Analysis of quantitative glycemic traits in fully developed diabetes is also complicated because most patients are taking glucose‐lowering medications, which involve different pharmacogenetic responses. Liu et al. investigated genome‐wide associations of prediabetes progression in the Atherosclerosis Risk in Communities (ARIC) study and the Framingham Heart Study (FHS). They identified 5 novel genes related to prediabetes, but failed to find any novel locus in single‐variant analyses [8]. Similarly, Lin et al. studied genetic associations of three diagnostic glucose traits and BMI in a Chinese population but found no novel loci below the nominal genome‐wide significance threshold of 5.0E‐08 [9].

Metabolomics has emerged as a powerful tool to reveal the underlying biological mechanisms underlying human health and disease [10]. Metabolomics offers the advantage of identifying small molecules that arise in the biological pathways of proteins encoded by human genes and elucidates metabolic byproducts arising from environmental factors such as dietary intake. Such an approach provides a more comprehensive snapshot for examining the integrated effects of multiple, often hierarchical, factors that contribute to disease development.

Glucose is derived from the metabolic breakdown of macronutrients — carbohydrates, fat, and protein — in our diet, making a healthy diet essential for lifestyle changes to prevent or reverse prediabetes. Previous studies have established associations between certain diet patterns and worsening glycemic control. Mediterranean, low‐fat, low‐glycemic index, vegetarian, and lower‐carbohydrate diets have been shown to be associated with improved glycemic control and better insulin sensitivity [11, 12, 13, 14]. However, the interactions between macronutrient intake and genetic contributions to metabolic pathways impacting glucose metabolism have yet to be widely studied.

This study aims to evaluate the genetic associations and gene‐macronutrient interactions of metabolites related to prediabetes.

## 2 Materials and Methods

We selected the cross‐sectional study design. We originally recruited 616 self‐reported Mexican American participants from Starr County, Texas in 2018‐2019 [15]. The exclusion criteria for this recruitment were individuals younger than 35 or older than 70 years old, any history of diagnosed diabetes, or use of glycemic‐lowering medications for any reason. Participants without genotype data and those taking glucose‐lowering medication for any reason were excluded, resulting in a final sample size of 482. Glycemic status groups was defined based on American Diabetes Association guidelines‐Normoglycemia group: individuals with a fasting plasma glucose <100 mg/dl and 2‐hour post‐load plasma glucose <140 mg/dl and HbA1c <5.7%(39 mmol/mol); Individuals with prediabetes: No diagnostic levels of diabetes and one of the following: fasting plasma glucose of 100–125 mg/dl, 2‐hour post‐load plasma glucose of 140–199 mg/dl, or HbA1c of 5.7–6.4% (39‐47 mmol/mol); Individuals with diabetes: fasting plasma glucose ≥ 126 mg/dl or 2‐hour post‐load plasma glucose ≥ 200 or HbA1c ≥ 6.5% (48 mmol/mol) [16]. In this sample, 113 individuals were classified with normal glycemia, 304 with prediabetes, and 65 with diabetes. To compare demographic characteristics across the glycemic groups, univariate ANOVA and chi‐square tests were used. Untargeted metabolomics data were produced by the Michigan Regional Comprehensive Metabolomics Research Core (MRC2). Initially, we selected metabolites with a missing rate of less than 50%. Afterward, all metabolites were inverse‐normalized. Macronutrient densities have been derived from a 106‐item food frequency questionnaire [17]. Detailed methods, including the manipulation of metabolomic data and nutrient densities, were described in our companion manuscript.

Generalized linear Mixed Model Association Tests (GMMAT) were used to investigate genetic variants affecting plasma metabolites, addressing cryptic relatedness and heterogeneity in the population structure [18]. We used GWAS array data imputed into the 1000 Genomes Project Phase 3 reference panel (n = 2,504) using the Michigan Imputation Server. Age, sex, and body mass index (BMI) were used as covariates. The kinship matrix was constructed using the GEMMA software [19]. Genetic data were converted into a PLINK bed file for analysis, and the minor allele frequency (MAF) was limited to a range of 0.01 to 0.5. Since metabolites are the quantitative traits, the GMMAT model was set up as a Gaussian family. We analyzed 308 named metabolites and 2,471 unnamed metabolites, with the significance threshold set at the nominal genome‐wide significance threshold of 5.0E‐08. Manhattan plots were generated by the R packages “qqman” and “topr”. For each significant association signal, the genomic position of each SNP was manually investigated using the University of California Santa Cruz(UCSC) Genome Browser to identify the nearest gene, based on the Human GRCh37/hg19 reference sequence [20]. R 4.2.2 version was used for the analysis.

Gene by environment (GxE) interaction analysis on metabolites by using nutrient density as environmental exposures was performed using Mixed Model Association Test for GEne‐Environment Interaction (MAGEE) that is also based on the generalized mixed model and adjusted for population structure using the same kinship matrix, making the analysis consistent with the previous analysis [21]. To minimize spurious associations caused by increased search space, we selected only the metabolites that showed significant associations with any macronutrients, glycemic and lipid traits, or with significant genetic associations in the GMMAT analysis. All the participants fasted overnight before blood sampling, and blood was drawn into EDTA tubes by trained staff at the field office. Glycemic traits included fasting glucose, 2‐hour post‐load glucose, HbA1c, insulin, and HOMA‐IR. HOMA‐IR is derived by the following formula: HOMA‐IR = fasting glucose in mmol/L*fasting insulin in µU/mL/22.5 [22]. Lipid traits included total cholesterol, high‐density lipoprotein (HDL), triglycerides, and non‐HDL cholesterol. Low‐density lipoprotein (LDL) was calculated by the Friedewald equation: LDL = Total cholesterol ‐HDL – (Triglycerides/5) [23]. Except for insulin and HOMA‐IR, all other glycemic and lipid traits were investigated and followed a normal distribution without extreme outliers; therefore, these traits were directly used for analysis to assess their association with metabolomics. HOMA‐IR and insulin levels were log‐transformed to account for high skewness. (Insulin: Shapiro‐Wilk test p‐value: <2.2E‐16; HOMA‐IR: Shapiro‐Wilk test p‐value:<2.2E‐16). The same number of metabolites associated with these traits, identified using linear regression models adjusted for age, sex, and BMI, with significance thresholds of p‐value < 1.62E‐04 for named metabolites and p‐value < 2.02E‐05 for unnamed metabolites, were described in our concurrent manuscript [24]. Therefore, 145 named metabolites and 687 unnamed metabolites were tested to find the gene‐nutrient intake interaction on metabolites. We used the same imputed GWAS data with the same MAF range of [0.01, 0.5]. MAGEE can generate p‐values for both interaction and joint tests. Interaction tests can assess the gene‐environment interaction, and joint tests can reveal the combined impact of the genetic main effect and the GxE interaction effect. Thus, we selected the significant signals based on the p‐value of the interaction test, with a threshold of 5.0E‐08, and the p‐value of the joint test, with a threshold of 5.0E‐07. We recognized that some QQ plots showed signs of deviation from neutral expectation; hence, we selected only the results with genomic control (λ_GC_) values between 0.95 and 1.15 to reduce false discoveries. R 4.2.2 version was used for the analysis.

## 3 Results

### 3.1 Demographics

Table 1 presents the demographics of the participants, categorized by their glycemic status, along with their total nutrient intake and nutrient densities. Although age was similar across the glycemic groups, the normal glycemia group had a significantly higher proportion of females (Chi‐square test p‐value 0.02). BMI was also significantly increased as worsening glycemia (univariate ANOVA p‐value: 6.25E‐12). However, the energy intake and nutrient densities of macronutrients did not show significant differences between the glycemic groups (p‐value > 0.05).

**TABLE 1:** Demographic characteristics of the participants.

Characteristics	Normal	Prediabetes	Diabetes
	(N = 113)	(N = 304)	(N = 65)
Age (years, Mean ± S.D.)	50.36 ± 7.32	51.34 ± 7.43	52.18 ± 7.56
Sex (no. (%))			
Female	94 (83.19)	213 (70.07)	49 (75.3)
BMI (kg/m^2^, Mean ± S.D.)	28.89 ± 5.12	32.66 ± 6.53	35.49 ± 7.11
Energy and Nutrient density^a^			
Energy (Kcal, Mean ± S.D.)	1963.19 ± 1093.56	2139.14 ± 1249.92	2018.83 ± 1140.84
Protein (%, Mean ± S.D.)	16.42 ± 5.19	17.15 ± 4.01	16.42 ± 3.51
Carbohydrates (%, Mean ± S.D.)	46.59 ± 10.85	45.35 ± 8.86	46.00 ± 8.01
Total Fat (%, Mean ± S.D.)	36.99 ± 7.65	37.50 ± 6.32	37.57 ± 6.12
Saturated Fat (%, Mean ± S.D.)	10.83 ± 2.51	11.11 ± 2.25	11.02 ± 1.90
Monounsaturated Fat (%, Mean ± S.D.)	15.47 ± 3.58	15.57 ± 2.89	15.68 ± 2.78
Polyunsaturated Fat (%, Mean ± S.D.)	7.42 ± 1.91	7.55 ± 1.76	7.63 ± 2.61

^a^ Nutrient density of each category was calculated as follows: [(Total nutrient intake(g) × W(g/kcal))/Total energy intake(kcal)] × 100; W was the weight decided by the nutrient categories‐ 4 in carbohydrates and protein, 9 in fat.

### 3.2 Genetic Variants Affecting Plasma Metabolites

#### 3.2.1 Named Metabolites

**FIGURE 1:**
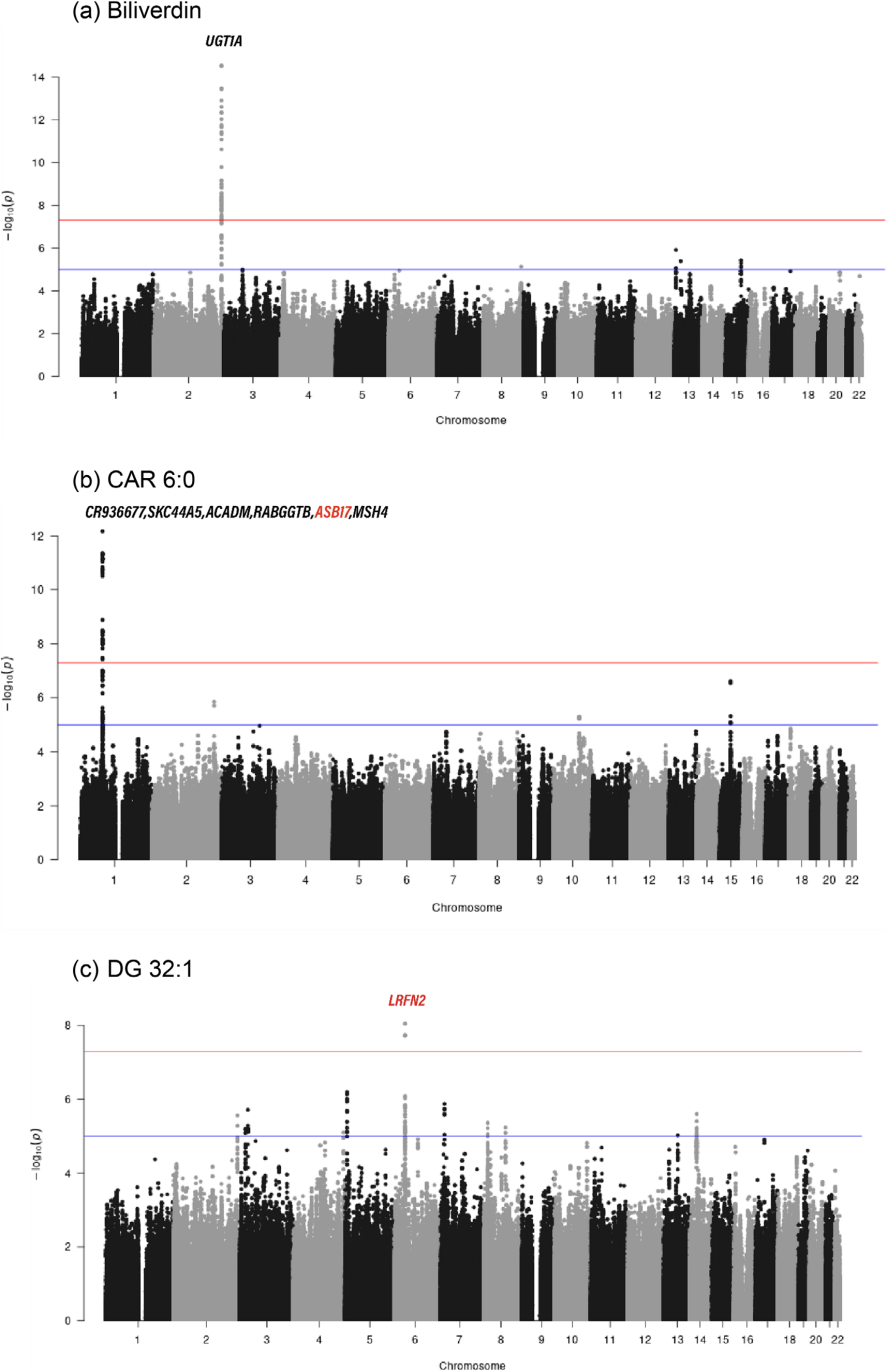
Examples of Manhattan plots of genetically associated identified metabolites. a) Genes highlighted in black represent previously established genetic associations, whereas those highlighted in red indicate the novel findings identified in this study. b) The red horizontal line is the significant p‐value threshold, 5.0E‐08, and c) the blue horizontal line is the suggestive p‐value threshold, 5.0E‐06.

A total of 28 metabolites showed statistically significant genetic associations with 1337 SNPs across 34 genes, as determined by GMMAT, after adjusting for age, gender, and BMI. The λ_GC_ values of significantly associated metabolites were between 0.970 and 1.015. 23 metabolite‐gene association pairs were repetitive from previous studies. Moreover, novel findings were identified between the identified metabolites and specific SNPs. Diacylglycerol (DG) 32:1 showed a significant signal(rs10947898) in the LRFN2 gene on chromosome 6, and Carnitine (CAR) 4:0 was associated with multiple SNPs in the five different genes, including *CABP1*. CAR 6:0 was known to have genetic associations with the SLAC44A5, ACADM, RABGGTB, and MSH4 genes, and a new association was identified at 1:76392437 (rs10732723) in the *ASB17* gene. There were additional novel genetic associations between variants in each gene and metabolite: *KANSL1L, RPE, UNC80, MYL1* genes‐CAR 9:0, *TGFA* gene‐ lysophosphatidylcholine (LPC) 20:0, *PHF21A, PEX17, LARGE2, PEX16* genes‐LPC 20:4_rp_b, *NRAS* gene ‐ lysophosphatidylethanolamine(LPE) 18:0_rp_a, *LARGE1* gene‐methyl‐3‐hydroxybenzoate, and *CAMSAP2* gene‐sphingosine. Table 2 summarizes the most significant genetic associations for named metabolites, and Figure 1 illustrates selected Manhattan plots.

**TABLE 2:** Summary of the most significantly genetically associated signals on identified metabolites and related genes.

Metabolite	SNP(GRCh37)	A1	A2	AF	SCORE	P‐value	Gene
Bilirubin	2:234664586	A	ATC	0.70	−104.42	3.78E‐13	*UGT1A*
Bilirubin	2:234665983	G	A	0.70	−104.42	3.78E‐13	*UGT1A*
Biliverdin	2:234664586	A	ATC	0.70	−117.16	2.96E‐15	*UGT1A*
Biliverdin	2:234665983	G	A	0.70	−117.16	2.96E‐15	*UGT1A*
CAR 10:1	1:76125211	A	C	0.71	82.80	2.88E‐09	*SLC44A5*
CAR 10:1	1:76159225	A	G	0.71	81.45	5.26E‐09	*CR936677*
CAR 10:1	1:76203479	AT	A	0.72	78.60	5.97E‐09	*ACADM*
CAR4:0	12:121176083	A	G	0.67	−191.87	4.67E‐41	*ACADS *
CAR4:0	12:121155622	T	C	0.71	−169.19	1.03E‐34	*UNC119B*
CAR4:0	12:121130046	G	A	0.70	−170.21	5.23E‐34	*MLEC*
CAR4:0	12:121084587	G	A	0.67	99.30	1.40E‐11	***CABP1 ***
CAR4:0	12:121200609	C	T	0.60	99.16	2.44E‐11	*SPPL3*
CAR4:0	12:121144144	C	G	0.58	88.91	1.59E‐08	—
CAR6:0	1:76168340	G	A	0.71	98.72	4.80E‐12	*CR936677*
CAR6:0	1:76106961	T	A	0.70	98.83	4.91E‐12	*SLC44A5*
CAR6:0	1:76192582	T	C	0.71	97.08	7.16E‐12	*ACADM*
CAR6:0	1:76259677	A	ACCTAAGAGTGAGACTTAACCCACTTTTAAATTGTTCT	0.73	84.08	1.29E‐09	*RABGGTB*
CAR6:0	1:76392437	C	T	0.74	81.37	4.79E‐09	***ASB17***
CAR6:0	1:76353294	C	T	0.72	78.31	3.78E‐08	*MSH4*
CAR8:0	1:76125211	A	C	0.71	84.60	1.47E‐09	*SLC44A5*
CAR8:0	1:76203479	AT	A	0.72	80.82	2.43E‐09	*ACADM*
CAR8:0	1:76159225	A	G	0.71	82.12	4.44E‐09	*CR936677*
CAR9:0	2:211074909	C	T	0.72	−134.98	1.82E‐23	*ACADL*
CAR9:0	2:211007287	C	T	0.66	−141.33	3.48E‐22	***KANSL1L***
CAR9:0	2:210878117	T	C	0.65	−132.89	4.09E‐20	***RPE***
CAR9:0	2:210846713	C	T	0.59	−117.31	9.58E‐15	***UNC80***
CAR9:0	2:211156513	T	TA	0.60	−118.27	2.79E‐14	***MYL1***
Cholic acid	9:98344706	T	A	0.83	63.23	2.97E‐08	—
DG 32:1	6:40532893	C	G	0.57	87.04	8.95E‐09	***LRFN2***
Dodecadienoic acid	4:149061102	T	C	0.97	30.20	3.93E‐08	***NR3C2***
Eicosatetraenoic acid	11:61603510	C	A	0.57	−85.28	2.61E‐09	* FADS2*
EPA	11:61609750	C	T	0.58	−78.56	4.37E‐08	* FADS2*
LPC20:0	2:70693652	C	A	0.84	53.48	2.78E‐08	***TGFA***
LPC20:4_rp_b	11:61603510	C	A	0.57	−151.44	1.43E‐25	* FADS2*
LPC20:4_rp_b	11:61537648	T	TG	0.76	−81.19	2.12E‐10	*MYRF*
LPC20:4_rp_b	11:46130135	TATAA	T	0.65	84.59	7.21E‐09	***PHF21A***
LPC20:4_rp_b	11:45939934	G	C	0.75	72.79	2.36E‐08	***PEX17***
LPC20:4_rp_b	1:45944186	TCGGGGG	T	0.75	72.79	2.36E‐08	***LARGE2***
LPC20:4_rp_b	11:45934549	AT	A	0.72	76.94	3.02E‐08	***PEX16***
LPE18:0_rp_a	1:115252909	TATAA	T	0.75	58.69	2.02E‐08	***NRAS***
LPE18:2_rp_b_1	11:61603510	C	A	0.57	95.17	4.37E‐11	* FADS2*
Methyl‐3‐hydroxybenzoate	22:34113903	C	A	0.85	64.3352	2.87E‐08	***LARGE1***
N‐Acetylleucine	2:73634622	TA	T	0.78	−74.26	3.23E‐12	*ALMS1*
N‐Acetylleucine	2:73867862	C	G	0.77	−74.53	1.15E‐11	*NAT8*
PC 34:2	11:61603510	C	A	0.57	87.88	1.42E‐09	* FADS2*
PC 36:2	11:46250265	GTAGA	G	0.64	79.27	3.94E‐08	—
PC 40:8	11:61603510	C	A	0.57	−116.08	5.30E‐16	* FADS2*
Pipecolic acid	4:181225524	T	C	0.62	−80.47	4.10E‐08	—
Sphingosine	1:200702431	C	T	0.59	95.30	7.89E‐10	***CAMSAP2***
Tetracosenoic acid	22:23786757	A	T	0.54	−87.31	2.27E‐08	—
1‐Methylxanthine	8:18272377	C	T	0.66	87.45	4.79E‐09	—
1,3‐Dimethyluric acid	13:112843248	T	C	0.63	−74.71	4.44E‐08	—
2‐Aminooctanoic acid	2:73805172	G	A	0.78	93.87	2.74E‐14	*ALMS1*
2‐Aminooctanoic acid	2:73867862	C	G	0.78	92.41	8.21E‐14	*NAT8*
2‐Aminooctanoic acid	2:73845709	T	C	0.57	99.68	7.40E‐12	—
2‐Aminooctanoic acid	2:73928366	G	C	0.56	94.10	1.97E‐10	*NAT8B*
2‐Aminooctanoic acid	2:73959960	G	T	0.57	91.23	7.66E‐10	*TPRKB*
2‐Hydroxybutyric acid	8:76334681	G	C	0.95	37.69	2.40E‐08	***HNF4G***
2‐Hydroxybutyric acid	8:76319588	A	G	0.95	35.71	4.89E‐08	—

^a^ All the analyses were done by GMMAT using score test, and all the analyses were adjusted for age, sex, and body mass index.

^b^ Boldly marked genes represent possible novel associations in this study, and all other genes were already known as the association with the matched metabolites.

^c^ Abbreviation: CAR carnitine; DG diacylglycerol; EPA eicosapentaenoic acid; LPC lysophosphatidylcholine; LPE lysophosphatidylethanolamine; PC phosphatidylcholine

#### 3.2.2 Unnamed Metabolites

A total of 232 unnamed metabolites showed significant genetic associations with 2,117 distinct SNPs by GMMAT after adjusting for age, gender, and BMI. From these 232 unnamed metabolites, 32 metabolites were associated with prediabetes status or glycemic traits, and 11 different genes were identified around the genomic variants associated with these metabolites. The list of these 32 metabolites and associated variants is summarized in Table S1. Although we do not know the identities of these unnamed metabolites, genetic association results provide clues about them. For example, variants located around *UGT1A* family genes in chromosome 2 were associated with UNK4, UNK9, UNK12, UNK13, UNK14, and UNK17. The same genomic region was also significantly associated with bilirubin and biliverdin, suggesting that these unnamed metabolites are closely related to the bilirubin and biliverdin pathways. Figure S1 illustrates example Manhattan plots of unnamed metabolites.

### 3.3 Interaction between Genetic Variation and Nutrient Intake on Plasma Metabolites

#### 3.3.1 Identified Metabolites

**FIGURE 2:**
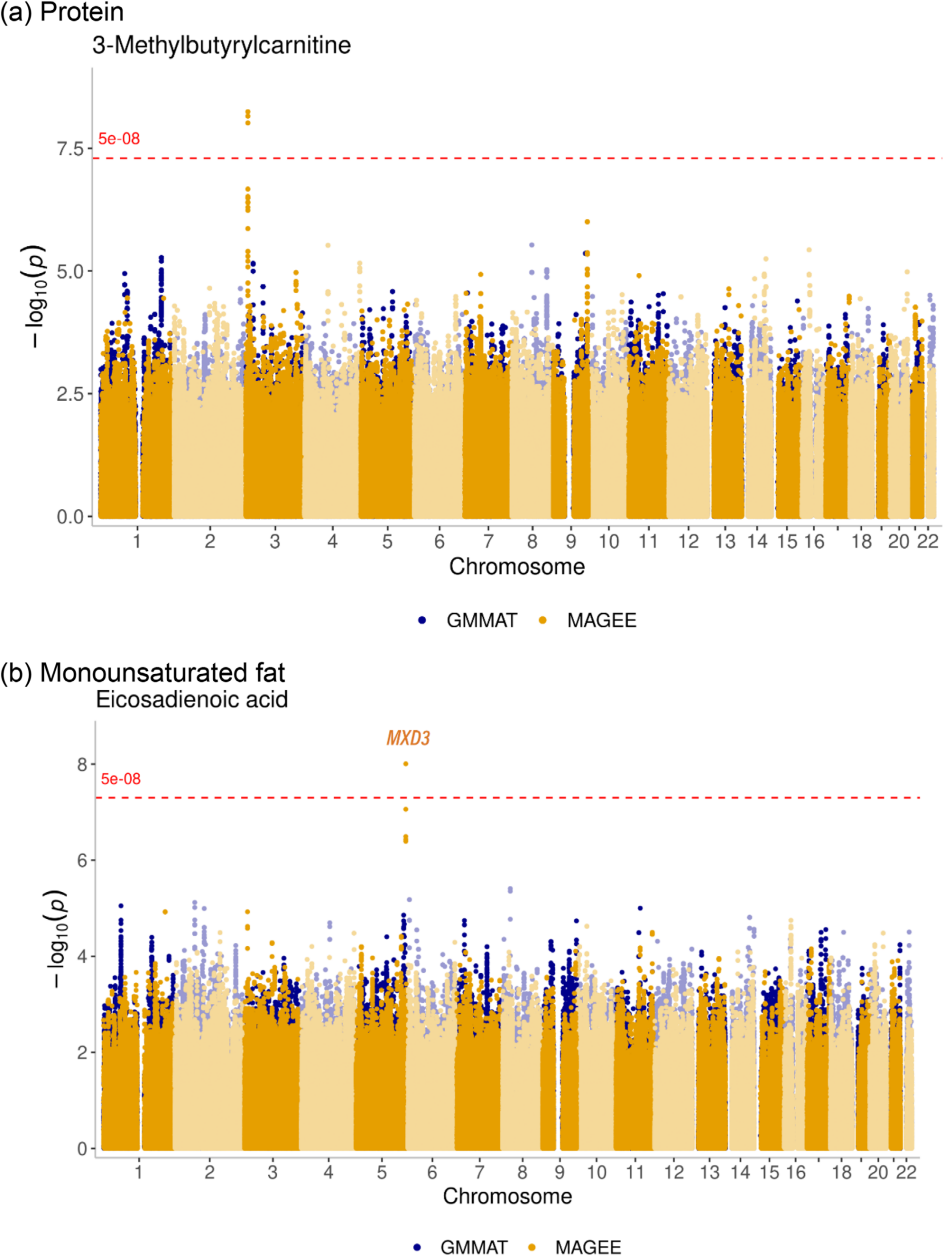
Example the Manhattan plots of the interaction p‐values for the significant gene‐nutrient interactions on identified metabolites by MAGEE compared to the results of GMMAT a) Blue dots were the p‐value of GMMAT on the same metabolite, and yellow dots were the p‐value of MAGEE, adding each nutrient category as the environmental interaction term.

To select the candidate metabolites, we analyzed the association between metabolites and glycemic traits, lipid traits, and nutrient intake, and the detailed results were described in our concurrent manuscript [24]. The list of metabolites associated with each trait is shown in Table S2. Carbohydrates, protein, total fat, saturated fat, monounsaturated fat, and polyunsaturated fat densities were tested separately to identify the gene‐nutrient interaction on 145 named metabolites, with age, gender, and BMI as covariates, using MAGEE. A total of 30 metabolites showed statistically significant interactions with each macronutrient nutrient density on 195 different variants. After filtering out results using genomic control (λ_GC_), we found significant interaction between 13 distinct variant and carbohydrate density on glutamic acid‐phenylalanine dipeptide (Glu‐Phe), 3 variants‐ protein density on 3‐methylbutyrylcarnitine, 3 different variants–protein density on phosphatidylcholine (PC) 38:6, 9 variants–saturated fat density on docosatetraenoic acid, 3 variants–saturated fat density on phenylacetic acid, one variant–monounsaturated fat density on eicosadienoic acid, and one variant–polyunsaturated fat density on 3‐Methyl‐2‐oxovaleric acid were identified to show variant–nutrient interactions on each metabolite, and total fat nutrient density did not have any significant interaction with single variants. Especially, one variant–protein density on 3‐methylbutyrylcarnitine, two variants–protein density on PC 38:6, and nine variants–saturated fat density on docosatetraenoic acid, one variant–monounsaturated fat density on eicosadienoic acid also showed statistical significance on variant–nutrient density interaction in the joint test with threshold 5.0E‐08. Interestingly, variants that showed significant interactions with nutrient densities did not exhibit significant genetic associations according to GMMAT in Section 3.2. Table 3 summarizes the named metabolites associated with variant‐nutrient density interaction results by MAGEE. Figure 2 shows example Manhattan plots showing the MAGEE interaction p‐values and GMMAT p‐values together for comparison.

**TABLE 3:** Summary of the lead signals on the identified metabolites with single variant‐nutrient density interaction.

Metabolites	Nutrient	SNP (GRCh37)	Effect Allele	AF	β G‐E	P‐Value Interaction	P‐Value Joint	GMMAT Score	GMMAT p‐value	Gene
Glu‐Phe	Carb.	19:20046102	A	0.03	0.122	1.36E‐08	9.84E‐08	0.973	0.823	*ZNF93*
3‐Methylbutyrylcarnit‐ine	Protein	3:6170316	T	0.37	−0.091	5.65E‐09	4.22E‐08	−2.677	0.870	—
PC38:6	Protein	6:129907587	T	0.26	−0.103	5.24E‐09	3.94E‐08	−1.457	0.917	*ARHGAP18*
Docosatetraenoic acid	Sat. Fat	10:1514015	T	0.64	−0.168	4.52E‐09	1.99E‐08	−15.058	0.295	*ADARB2*
Phenylacetic acid	Sat. Fat	3:53345623	C	0.24	−0.180	2.74E‐08	1.89E‐07	−3.859	0.778	*DCP1A*
Eicosadienoic acid	Mon. Fat	5:176735612	G	0.33	0.131	9.88E‐09	2.84E‐08	20.313	0.162	*MXD3*
3‐Methyl‐2‐oxovaleric acid	Poly. Fat	3:55577710	A	0.71	−0.198	3.03E‐08	1.63E‐07	−12.220	0.437	*ERC2*

^a^ β G‐E, p‐value Interaction, and p‐value Joint were calculated by MAGEE. The GMMAT score and GMMAT p‐value were calculated by GMMAT to assess the genetic association between the same single variant and its matched metabolite. AF represents the effect allele frequency. GMMAT and MAGEE used the same covariates: age, gender, and BMI.

^b^ Abbreviation: Carb: Carbohydrate; Sat.Fat: Saturated Fat; Mon.Fat: Monounsaturated Fat; Poly. Fat: Polyunsaturated Fat; PC: Phosphatidylcholine.

#### 3.3.2 Unnamed Metabolites

We selected 687 unnamed metabolites that showed associations with glycemic traits, lipid traits, macronutrients, or genetic association signals to identify gene‐nutrient interactions. We identified 32 unnamed metabolites with significant gene‐nutrient interaction analyses after the same λ_GC_‐based filtering. There were 39 variant‐carbohydrate interaction pairs associated with seven distinct unnamed metabolites, 11 variant‐protein interaction pairs on six metabolites, 58 variant‐total fat interaction pairs on five metabolites, 17 variant‐saturated fat interaction pairs on five metabolites, 38 variant‐monounsaturated fat interaction pairs on five metabolites, and 20 variant‐polyunsaturated fat interaction pairs on four metabolites. A single variant at 1:14463067(rs6671072) interacted with total fat and monounsaturated fat on UNK37 that was associated with log‐transformed insulin and log‐transformed HOMA‐IR [24]. 14 variants in the same region also showed significant interactions with total fat and monounsaturated fat on UNK36, and this unnamed metabolite was associated with 2‐hour post‐load glucose, log‐transformed insulin, log‐transformed HOMA‐IR, HDL, triglyceride, and prediabetes categories in our previous analysis [24]. 14 unnamed metabolites having genetic associations also showed significant GxE interactions with nutrient densities, but no variants overlapped between GMMAT and MAGEE results. 25 variant‐carbohydrate interaction pairs on UNK33, one variant‐protein interaction pair on UNK34, 12 variant–saturated fat interaction pairs on UNK35, one variant‐total fat interaction pair, and one variant‐saturated fat interaction pair on UNK1 also showed significant interaction results with joint tests. Table S3 summarizes the most significant signals on one or two different nutrient interaction analyses by MAGEE. Figure S2 shows the example Manhattan plots of gene‐nutrient interaction on unnamed metabolites.

## 4 Discussion

We identified 28 genetic associations with named metabolites and 232 genetic associations with unnamed metabolites using a p‐value threshold of < 5.0E‐08. Among the 28 named metabolites, biliverdin, CAR 4:0, DG 32:1, eicosatetraenoic acid, eicosapentaenoic Acid (EPA), N‐acetylleucine, and 2‐hydroxybutyric acid were associated with glycemic traits, CAR 6:0, DG 32:1, eicosatetraenoic acid, LPC 10:4_rp_b, LPE 18:0_rp_a, PC 34:2, and pipecolic acid were associated with lipid traits, and LPC 20:0 and 2‐aminooctanoic acid were associated with BMI only, with p‐value threshold of <1.62E‐04 in our concurrent manuscript [24]. We also discovered a novel genetic association between a variant located in the *LRFN2* gene (rs10947898) and DG 32:1 that has not been previously reported. The *LRFN2* gene encodes a synaptic cell adhesion protein in the brain and is related to memory deficits and Alzheimer's disease [25]. Interestingly, this gene is also expressed in pancreatic islet cells and is associated with insulin secretion [26, 27]. In another concurrent manuscript, DG 32:1 was positively associated with log‐transformed insulin and HOMA‐IR [24]. Thus, the expression of *LRFN2* can impact insulin secretion, and this could also affect the DG 32:1 in the blood. Multiple SNPs on *the LRFN2* gene have been repeatedly reported to be related to type 2 diabetes, BMI, waist‐hip ratio, visceral adipose tissue adjustment, triglycerides, HDL, and non‐HDL levels [28, 29, 30, 31, 32, 33, 34, 35, 36, 37, 38, 39, 40] Thus, the novel finding about the genetic association of the variant on *LRFN2* with DG 32:1 could be a clue to how this gene impacts those phenotypes through metabolic pathways involving DG 32:1. We identified 33 variant‐nutrient interactions significantly associated with named metabolites and 183 variant‐nutrient interactions associated with unnamed metabolites. Among these variant‐nutrient interaction pairs, 13 pairs for named metabolites and 40 pairs for unnamed metabolites also showed significant results (P<5.0E‐08) on the joint tests. Interestingly, we initially did not identify any significant genetic association with eicosadienoic acid (GMMAT p‐value = 0.16). However, the joint test revealed a significant association with the MXD3 gene when we added the nutrient density of monounsaturated fat as an interaction term (interaction p‐value 9.9E‐09 and joint p‐value 2.8E‐08). The *MXD3* gene is a transcription factor that has been previously reported to be associated with obesity, and it can promote the lipid‐related pathway, thereby impacting visceral adiposity [41]. Intake of monounsaturated fat can interfere with these lipid metabolism pathways.

However, none of the same SNPs exhibited significant results in both GMMAT and MAGEE with the p‐value threshold 5E‐08. Thus, this study suggests that variants weakly genetically associated with specific metabolites by GMMAT may have stronger gene‐nutrient interactions; however, additional studies are needed to support these findings.

All the participants of our study are self‐reported Mexican Americans, a genetically understudied but fast‐increasing population in the U.S, with a growing prevalence of cardiometabolic disorders [42, 43]. Fifty‐three percent of the participants in this study reported having no health insurance, and more than half of the participants answered that their household incomes were below $30,000 per year [44]. Thus, the economic burden resulting from worsening glycemia is substantially greater among these low‐income populations, who are more vulnerable to the financial consequences of disease progression, compared with higher‐income populations that generally have greater access to healthcare resources and protective socioeconomic factors. Combined analyses of nutrient intake, metabolomics, and genetics in this study could provide crucial evidence to develop strategies toward further large‐scale longitudinal studies that could provide a mechanistic understanding of how genetics and nutrient intake interact under dietary interventions targeting this population [45] Another strength of this study is that we could identify several novel genetic associations for metabolites despite the moderate sample size, partially empowered by the uniqueness of the study population. Among the named metabolites, genetic associations for CAR 9:0, DG 32:1, dodecadienoic acid, LPC 20:0, LPC 20:4, LPE 18:2, sphingosine, and 2‐hydroxybutyric acid have not been previously described. We also successfully replicated previously identified genetic associations for bilirubin, biliverdin, CAR 10:1, CAR 4:0, CAR 6:0, CAR 8:0, Eicosatetraenoic acid, EPA, N‐Acetylleucine, PC 34:2, PC 40:8, 2‐aminooctanoic acid, reassuring the technical stability and validity of our findings. To the best of our knowledge, this is the first study to investigate the association between gene‐nutrient interactions and metabolites in Mexican Americans. Previous gene‐environmental interaction studies typically tested specific target genomic regions; however, this study investigated genome‐wide gene–nutrient interactions using imputed whole‐genome data. Notably, most variants with significant gene‐nutrient interactions did not exhibit a strong enough effect through genetic association analysis alone, emphasizing the need to assess the attribution of environmental factors in genetic analyses of metabolites.

Still, this study has some limitations. First, our statistical power is limited due to a moderate sample size, and there were additional exclusions due to medication use and lack of genomic data, which could affect stability in MAGEE results, because the method was originally developed for the large sample‐size investigation of more than 2000 [21] In the follow‐up studies, we will utilize our longitudinal metabolomic data to replicate and validate our findings. Another limitation is that recall bias could compromise the macronutrient data from the food frequency questionnaire [15]. Although the participants were carefully instructed about their food frequency questionnaire to decrease the impact of recall bias, a certain extent of recall bias would still be inevitable in the nutrient study, given that the questionnaire was used to assess their food frequency. To minimize this bias, the questionnaire has been previously validated in this population and is the best available source of information for food intake [17]. Last, the identification of unnamed metabolites would provide further insights into the biology of prediabetes and the interaction between genes and nutrients; hence, we will prioritize several unnamed metabolites and perform additional molecular and informatics analyses to reveal their identities. At this stage, we checked the correlation between named and unnamed metabolites, and the supplementary tables included the most correlated named metabolites to provide additional information.

In conclusion, we identified multiple genetic associations with 28 named and 232 unnamed metabolites, including variants in the LRFN2 gene that were associated with DG 32:1, a metabolite linked to carbohydrate, total fat, and monounsaturated fat intakes. We also identified seven significant variant‐macronutrient density interaction pairs among named metabolites; however, there was no overlap between variants from the genetic association analyses and those identified through the interaction analyses, suggesting the need for further follow‐ups to pinpoint causal signals and pathways.

## Funding

This study was supported by the National Institutes of Health/National Institute of Diabetes and Digestive and Kidney Diseases, R01DK118631, R01DK109920, and R01DK116378. Shinhye Chung was also supported as a predoctoral fellowship by the National Institute of Environmental Health Sciences, T32ES027801.

## Ethics Approval Statement

All the participants in this study participated voluntarily, and informed consent was obtained as approved by the institutional review board (IRB).

## Conflicts of Interest

HC receives consulting fees from Character Biosciences. The authors affirm that no relationships or activities exist that could influence or be perceived as influencing the integrity of this article.

## Supporting information




**Supporting File**: ggn270020‐sup‐0001‐Figure S1‐S2.docx.

**Supporting File**: ggn270020‐sup‐0001‐Table S1–S3.docx.

## Data Availability

Genotype and phenotype data are available from dbGaP (study accession phs001166). Additional data are available on request from the corresponding author. The data are not publicly available due to privacy or ethical restrictions.
