# Supplementary material for: Metabolomic Signatures of Prediabetes in Mexican Americans: The Role of Genetics and Macronutrients
Source: Adv Genet (Hoboken). 2025 Dec 16;6(4):e00032. doi: 10.1002/ggn2.202500032 (PMC12747543; doi:10.1002/ggn2.202500032)

Supplementary figure 1. Examples of Manhattan plots of genetically associated unidentified metabolites

1. UNK12: associated with log-transformed insulin and log-transformed HOMA-IR


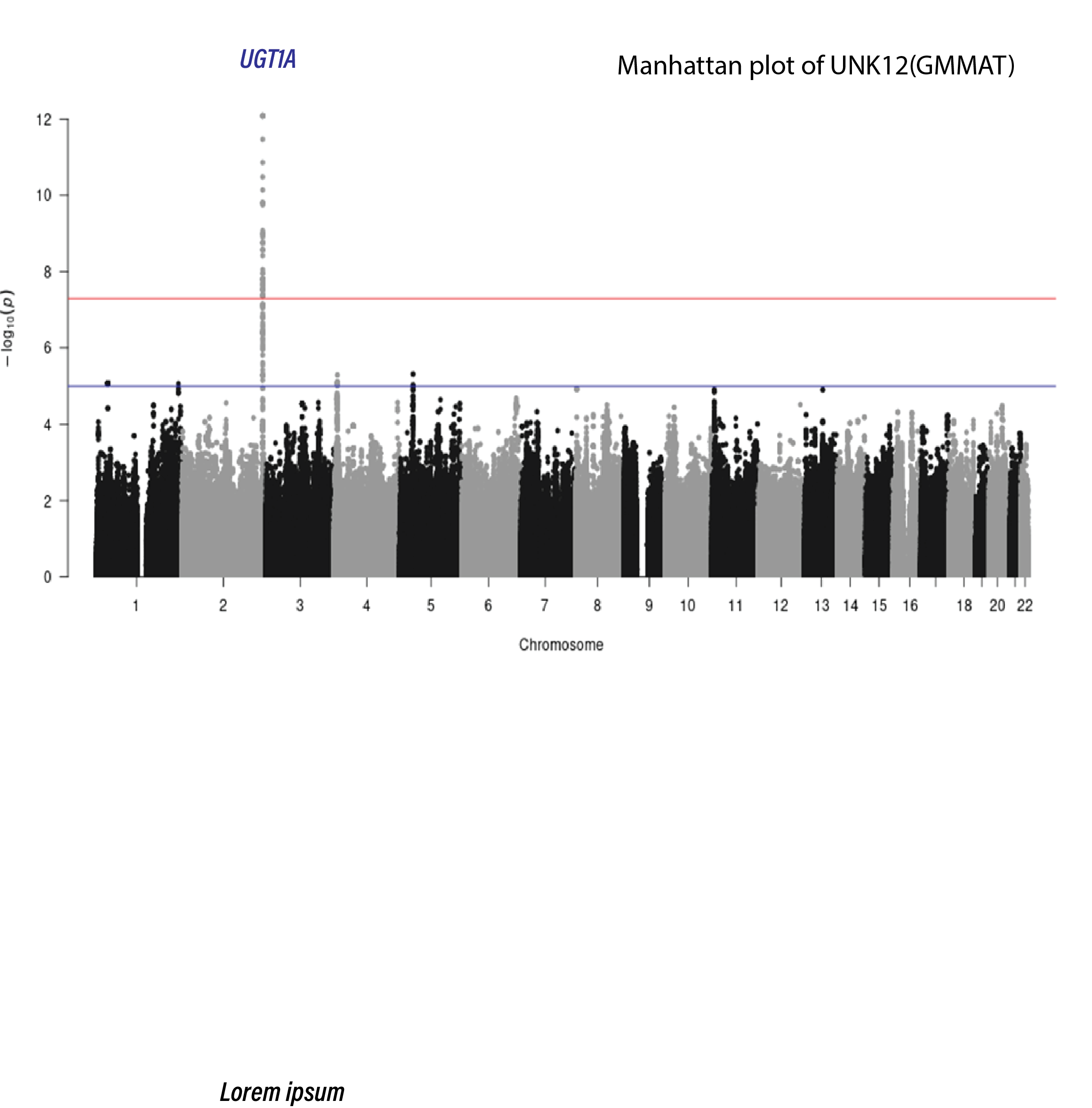


1. UNK10: associated with 2-hour post-load glucose


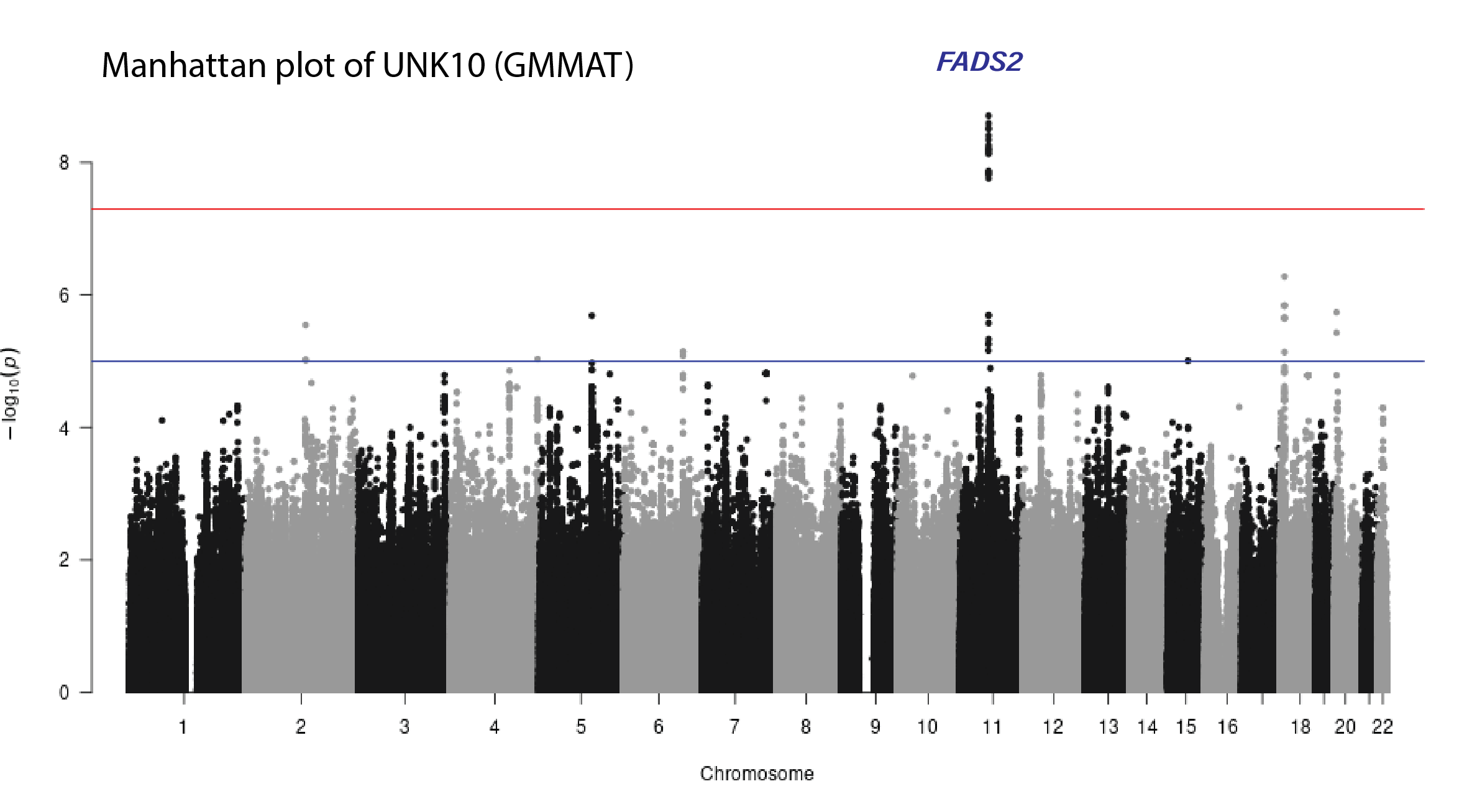


1. UNK11: associated with fasting plasma glucose, HbA1c, log-transformed insulin, log-transformed HOMA-IR, groups with different glycemic groups by the linear model, the group with non-diabetes vs. the group with diabetes, and triglyceride


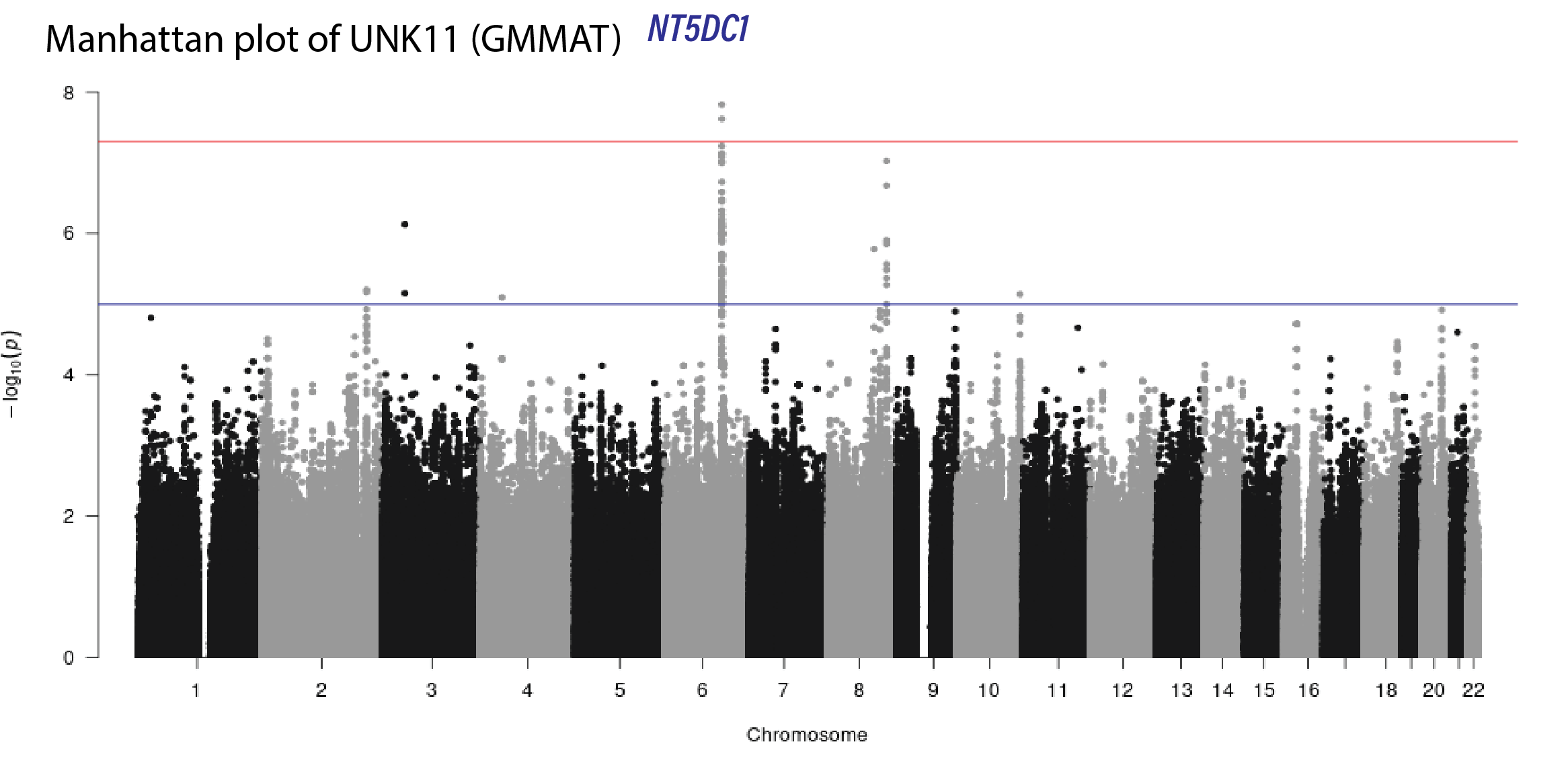


1. UNK24: associated with 2-hour post-load glucose, groups with different glycemic groups by the linear model, normal Vs. the group with diabetes, normal Vs. the group with prediabetes, normal Vs. the group with hyperglycemia, the group with non-diabetes Vs. the group with diabetes, and BMI


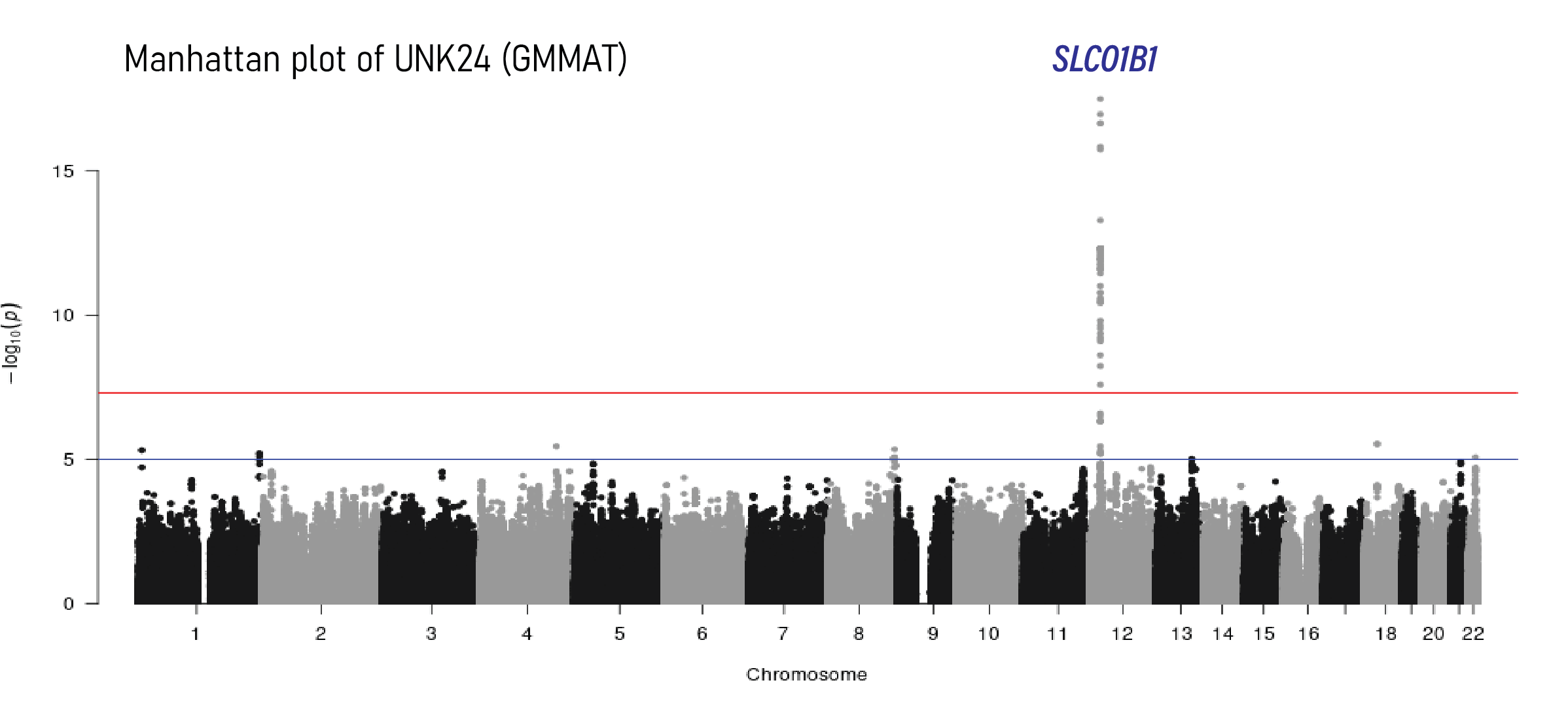


Supplementary figure 2. Example Manhattan plots of the interaction p-values for the significant gene-nutrient interactions on unidentified metabolites by MAGEE compared to the results of GMMAT

1. Carbohydrate on UNK33


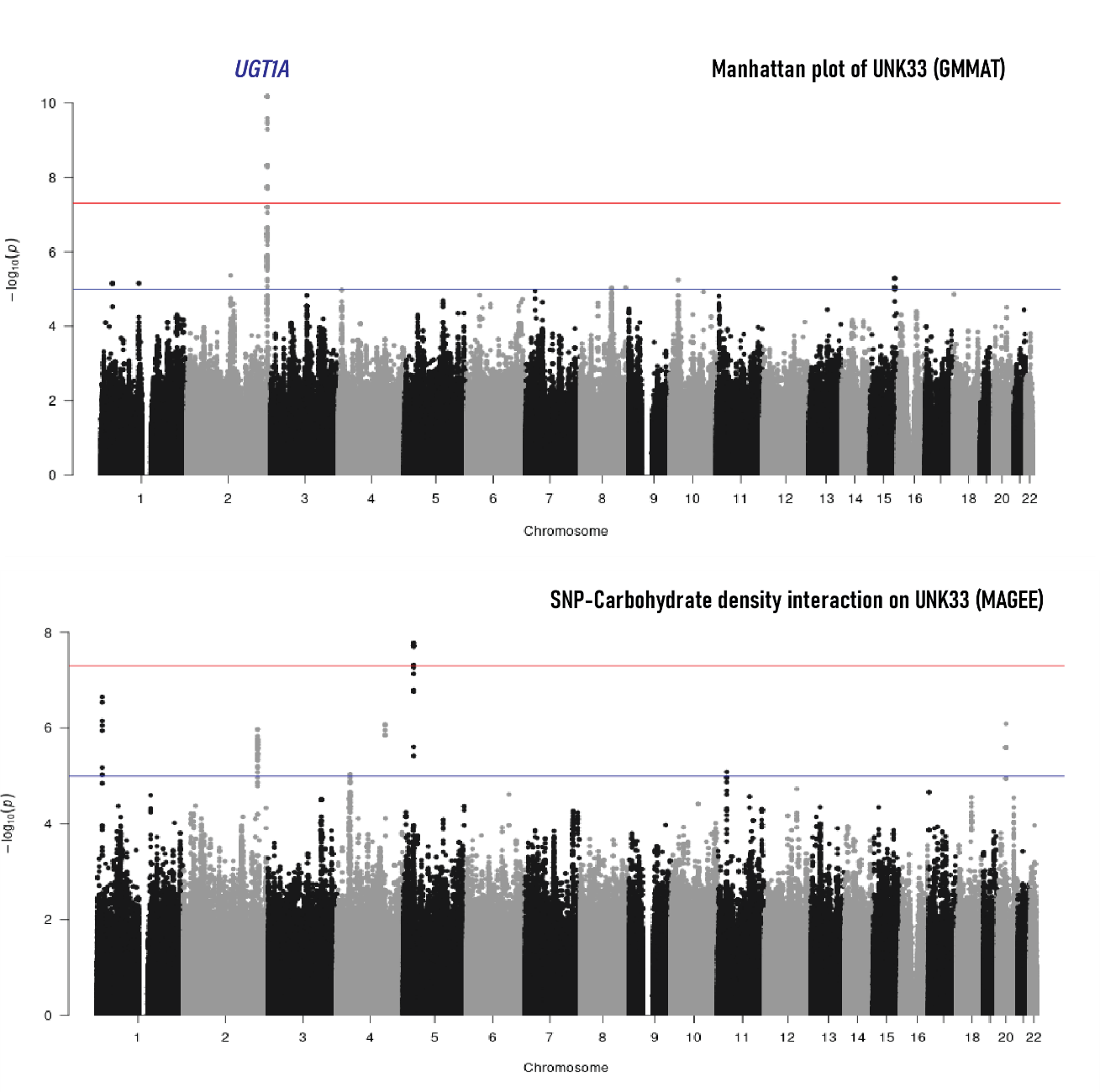


1. Protein on UNK34


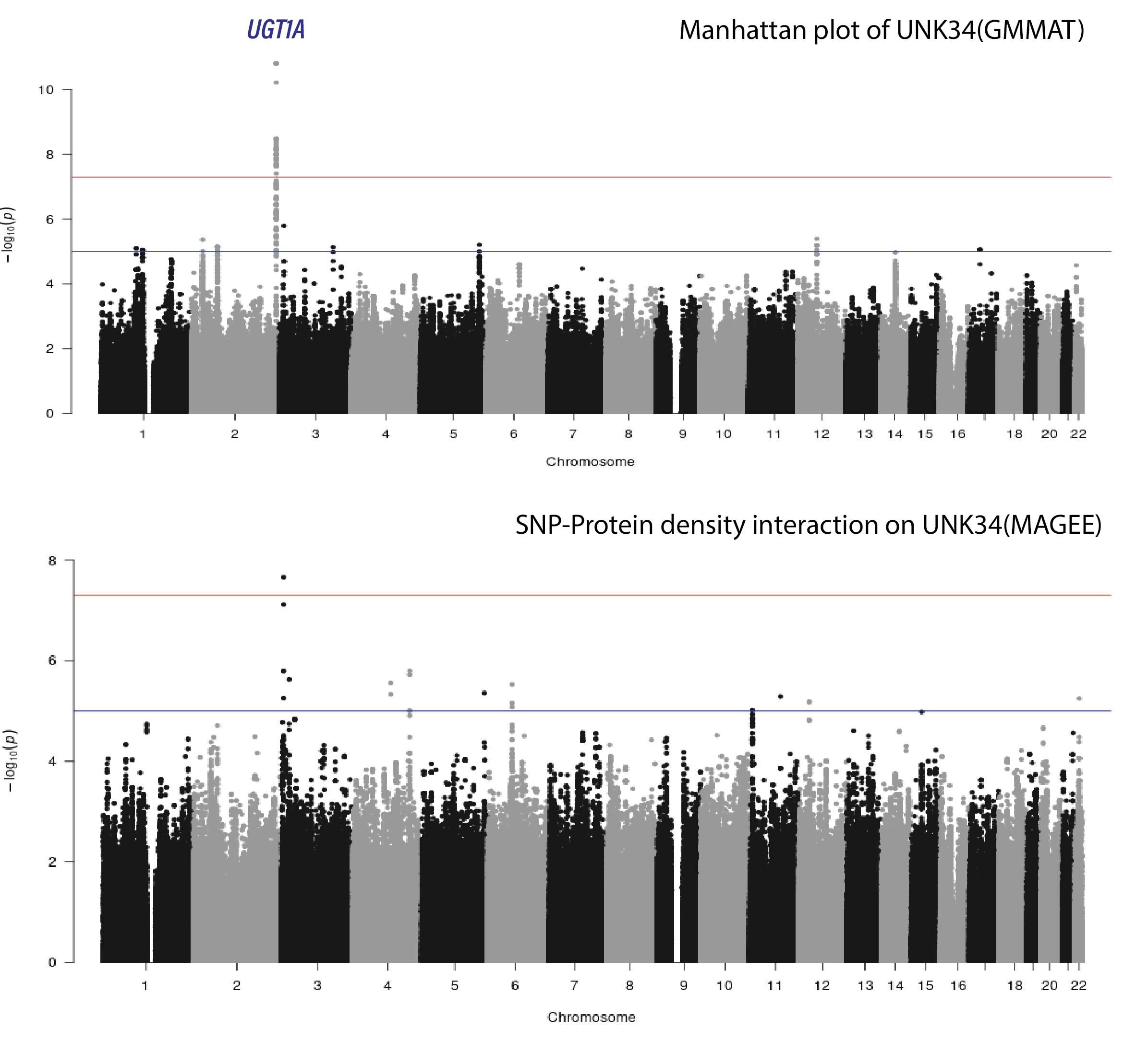


1. Saturated fat on UNK35


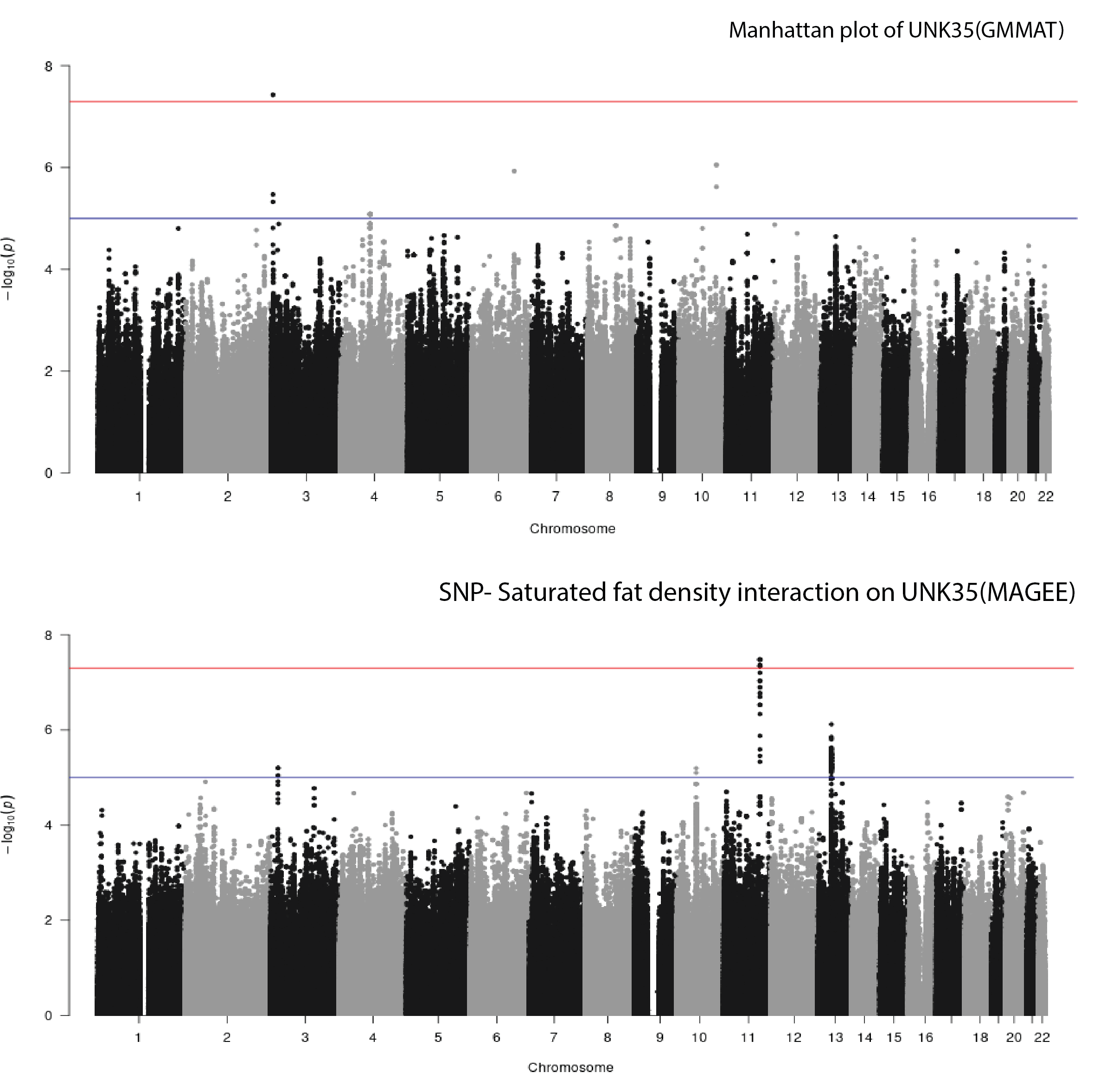


1. Saturated fat on UNK1


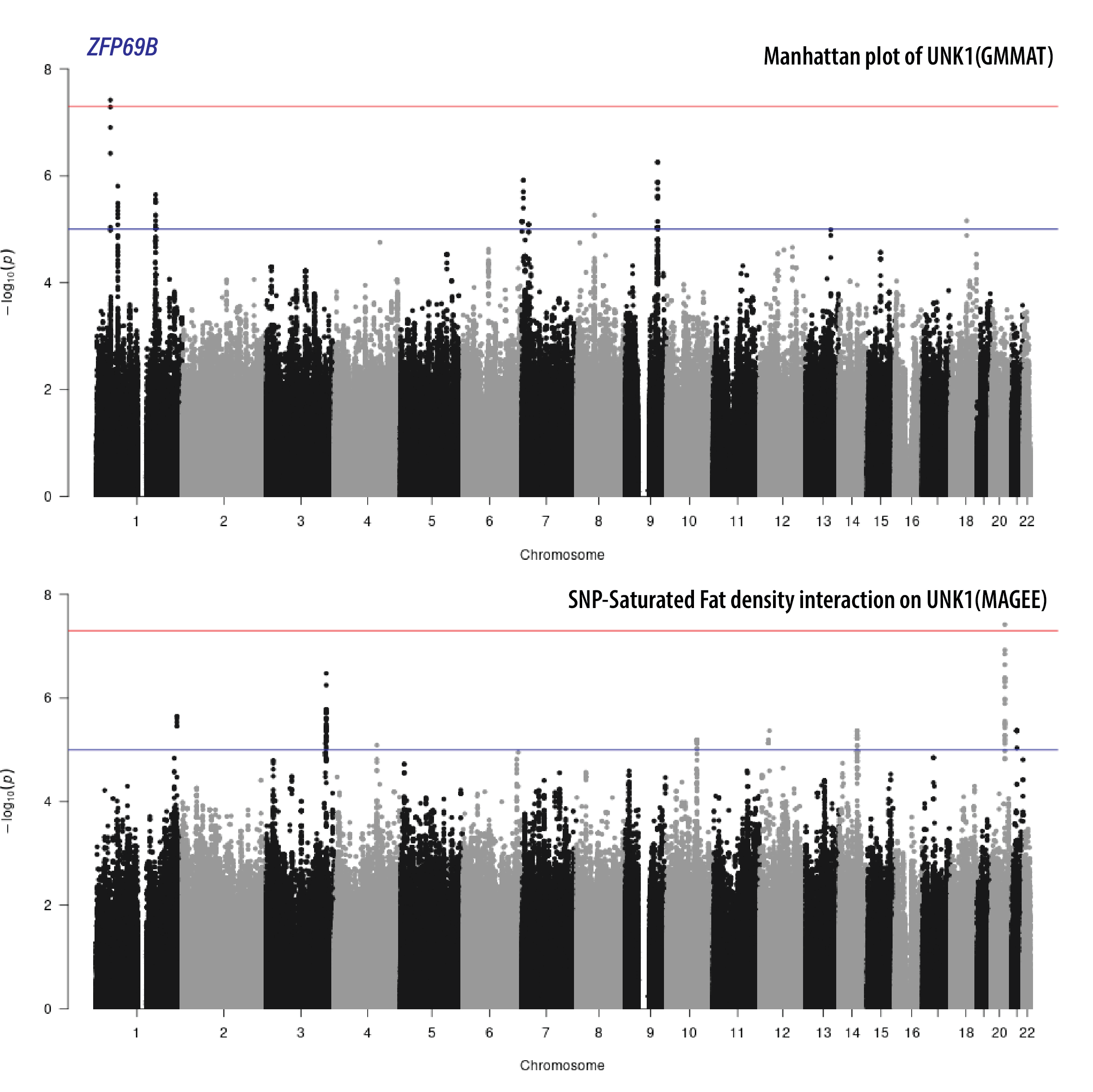


1. Total fat and monounsaturated fat on UNK36


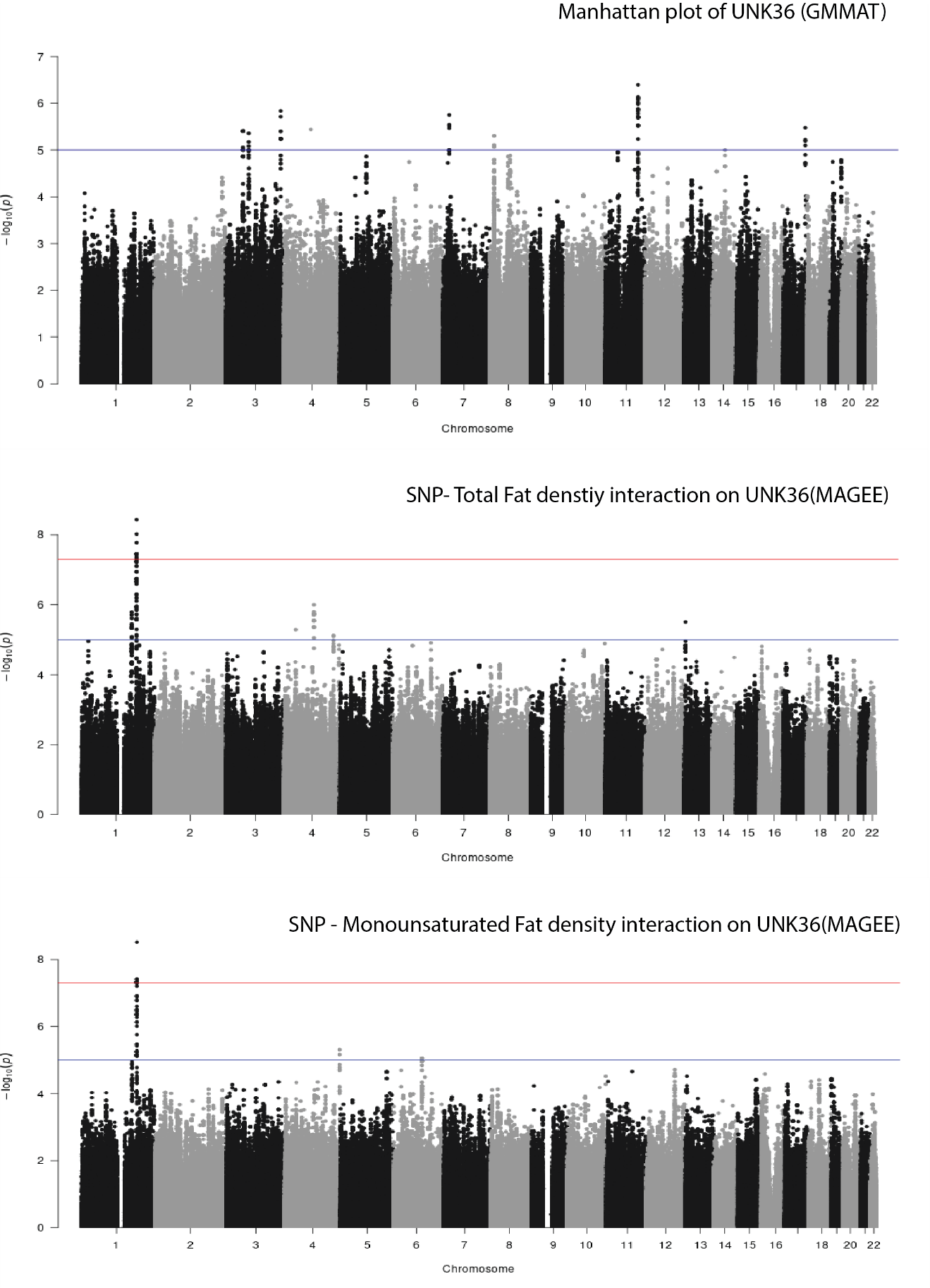


1. Total fat on monounsaturated fat on UNK37


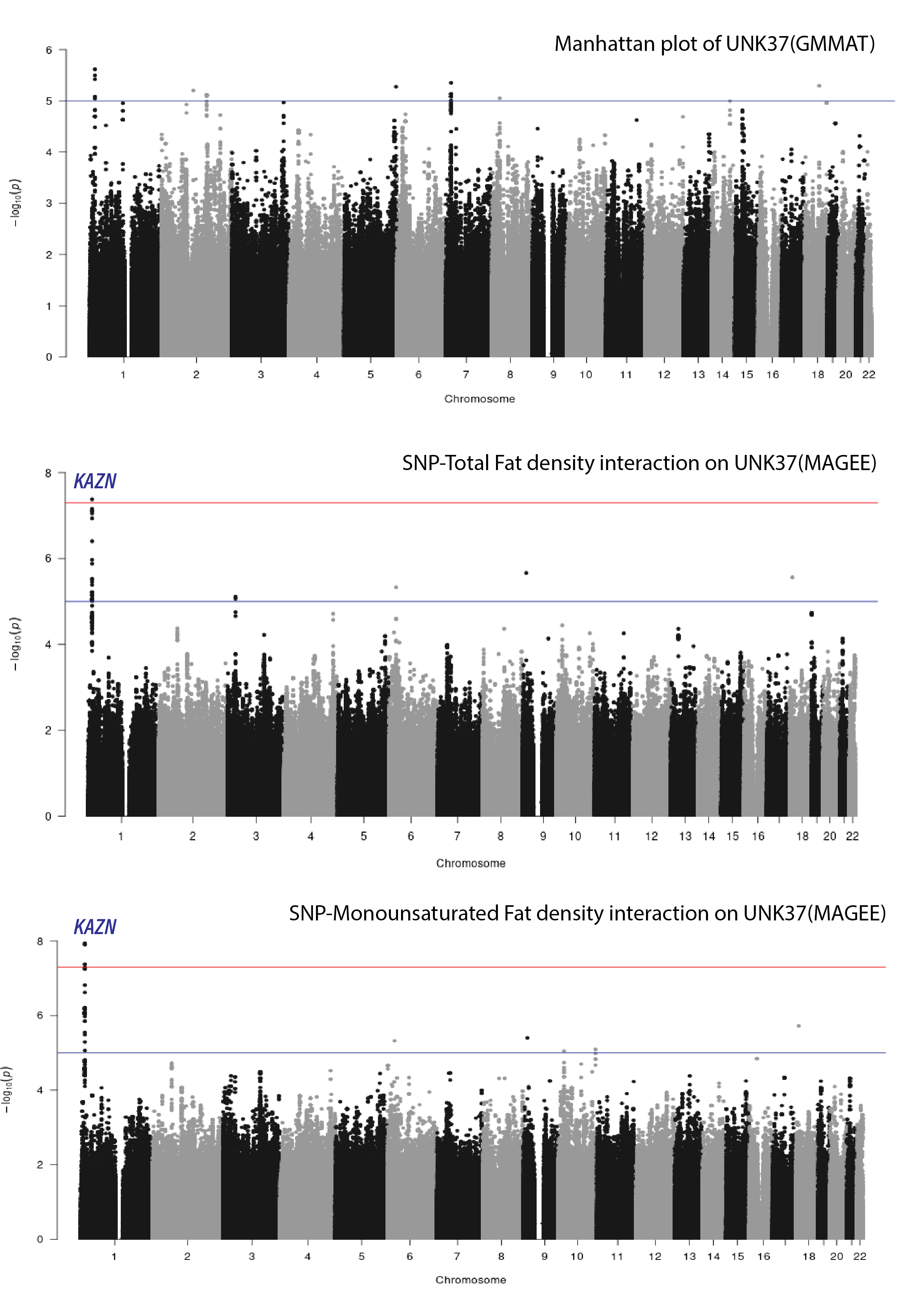

Supplement: Supplementary file 1 — Supporting File: ggn270020‐sup‐0001‐Figure S1‐S2.docx. [file GGN2-6-e00032-s002.docx]
