# Supplementary material for: Metabolomic Signatures of Prediabetes in Mexican Americans: The Role of Genetics and Macronutrients
Source: Adv Genet (Hoboken). 2025 Dec 16;6(4):e00032. doi: 10.1002/ggn2.202500032 (PMC12747543; doi:10.1002/ggn2.202500032)
Supplement: Supplementary file 2 — Supporting File: ggn270020‐sup‐0001‐Table S1–S3.docx. [file GGN2-6-e00032-s001.docx]

Supplementary table 1. Summary of lead single variant signals of unnamed metabolites and related genes

| Unnamed Metabolites | Most correlated named metabolites | SNP | | A1 | | A2 | AF | SCORE | VAR | p-value | Gene |
| --- | --- | --- | --- | --- | --- | --- | --- | --- | --- | --- | --- |
| UNK1 | Octadecadienoic acid  (Corr 0.74) | | 1:40922240 | | C | T | 0.65 | 87.90 | 255.493 | 3.81E-08 | *ZFP69B* |
| UNK2 | Hydroxydecanoic.acid (Corr:0.65) | | 18:58392436 | | A | G | 0.51 | -87.58 | 247.574 | 2.60E-08 | *-* |
| UNK3 | N-Acetylcerine  (Corr: 0.99) | | 4:10005435 | | G | A | 0.74 | 85.57 | 233.877 | 2.20E-08 | *SLC2A9* |
| UNK4 | Biliverdin  (Corr: 0.80) | | 2:234664586 | | A | ATC | 0.70 | -82.36 | 213.214 | 1.70E-08 | *UGT1A* |
|  |  |  | 2:234665983 | | G | A | 0.70 | -82.36 | 213.214 | 1.70E-08 | *UGT1A* |
| UNK5 | Nonadecenoic acid  (Corr: 0.63) | | 7:26381618 | | C | T | 0.82 | -69.50 | 159.092 | 3.58E-08 | *SNX10* |
| UNK6 | Eicosatetraenoic acid  (Corr: 0.94) | | 11:61603510 | | C | A | 0.57 | -90.73 | 205.622 | 2.49E-10 | *FADS2* |
| UNK7 | Heptadecanedioic.acid  (Corr: 0.89) | | 12:21331549 | | C | T | 0.86 | -58.96 | 112.344 | 2.66E-08 | *SLCO1B1* |
| UNK8 | None | | 6:132711678 | | G | T | 0.88 | 54.48 | 97.5177 | 3.45E-08 | *MOXD1* |
| UNK9 | Biliverdin  (Corr: 0.77) | | 2:234664586 | | A | ATC | 0.70 | -98.22 | 219.423 | 3.33E-11 | *UGT1A* |
|  |  |  | 2:234665983 | | G | A | 0.70 | -98.22 | 219.423 | 3.33E-11 | *UGT1A* |
| UNK10 | EPA(Corr: 0.87) | | 11:61609750 | | C | T | 0.58 | -88.59 | 217.889 | 1.95E-09 | *FADS2* |
| UNK11 | MG (16:0)_rp_a  (Corr: 0.75) | | 6:116491302 | | A | G | 0.74 | -74.37 | 172.554 | 1.50E-08 | *NT5DC1* |
| UNK12 | None | | 2:234664586 | | A | ATC | 0.70 | -105.90 | 218.973 | 8.27E-13 | *UGT1A* |
|  |  | | 2:234665983 | | G | A | 0.70 | -105.90 | 218.973 | 8.27E-13 | *UGT1A* |
| UNK13 | Biliverdin  (Corr: 0.91) | | 2:234664586 | | A | ATC | 0.7 | -114.28 | 220.268 | 1.36E-14 | *UGT1A* |
|  |  |  | 2:234665983 | | G | A | 0.7 | -114.28 | 220.268 | 1.36E-14 | *UGT1A* |
| UNK14 | Biliverdin  (Corr: 0.90) | | 2:234664586 | | A | ATC | 0.70 | -110.89 | 219.972 | 7.64E-14 | *UGT1A* |
|  |  |  | 2:234665983 | | G | A | 0.70 | -110.89 | 219.972 | 7.64E-14 | *UGT1A* |
| UNK15 | LPC(20:4)_rp_b  (Corr: 0.62) | | 11:61603510 | | C | A | 0.57 | -86.74 | 208.554 | 1.89E-09 | *FADS2* |
|  |  |  | 11:44712698 | | C | G | 0.75 | 74.91 | 179.21 | 2.20E-08 | *-* |
| UNK16 | N-Acetylserine  (Corr: 0.95) | | 4:10004805 | | C | T | 0.74 | 86.76 | 243.848 | 2.76E-08 | *SLC2A9* |
| UNK17 | Biliverdin  (Corr: 0.83) | | 2:234664586 | | A | ATC | 0.70 | -106.33 | 217.123 | 5.35E-13 | *UGT1A* |
|  |  |  | 2:234665983 | | G | A | 0.70 | -106.33 | 217.123 | 5.35E-13 | *UGT1A* |
| UNK18 | Eicosatetraenoic acid  (Corr: 0.84) | | 11:61603510 | | C | A | 0.57 | -95.70 | 207.84 | 3.18E-11 | *FADS2* |
| UNK19 | Eicosatetraenoic acid  (Corr: 0.89) | | 11:61603510 | | C | A | 0.57 | -89.84 | 202.362 | 2.70E-10 | *FADS2* |
|  |  |  | 11:44712698 | | C | G | 0.75 | 72.34 | 175.805 | 4.87E-08 | *-* |
| UNK20 | Eicosatetraenoic acid  (Corr: 0.78) | | 11:61603510 | | C | A | 0.57 | -103.92 | 198.627 | 1.67E-13 | *FADS2* |
| UNK21 | None | | 12:21331549 | | C | T | 0.86 | -74.82 | 116.914 | 4.51E-12 | *SLCO1B1* |
|  |  | | 12:21399955 | | A | G | 0.86 | -67.33 | 116.91 | 4.75E-10 | *-* |
| UNK22 | Leucine-Isoleucine  (Corr: 0.90) | | 3:189534621 | | T | C | 0.72 | 90.59 | 256.163 | 1.51E-08 | *TP63* |
| UNK23 | γ-glytamylisoleucine  (Corr: -0.55) | | 12:4940628 | | A | G | 0.88 | 63.44 | 130.829 | 2.91E-08 | *GALNT8* |
| UNK24 | None | | 12:21331549 | | C | T | 0.86 | -93.82 | 116.281 | 3.31E-18 | *SLCO1B1* |
|  |  | | 12:21399955 | | A | G | 0.86 | -88.88 | 116.61 | 1.86E-16 | *-* |
| UNK25 | None | | 12:21331549 | | C | T | 0.86 | -89.67 | 117.483 | 1.30E-16 | *SLCO1B1* |
| UNK26 | None | | 12:21331549 | | C | T | 0.86 | -64.23 | 119.252 | 4.07E-09 | *SLCO1B1* |
| UNK27 | None | | 12:21331549 | | C | T | 0.86 | -87.83 | 117.545 | 5.44E-16 | *SLCO1B1* |
|  |  | | 12:21399955 | | A | G | 0.86 | -83.10 | 117.686 | 1.86E-14 | *-* |
| UNK28 | LPE (18:0)_rp_a  (Corr: 0.72) | | 15:58726744 | | G | C | 0.56 | -98.07 | 241.358 | 2.74E-10 | *-* |
| UNK29 | LPC (20:4)_rp_a  (Corr: 0.75) | | 11:61603510 | | C | A | 0.57 | -92.93 | 203.218 | 7.09E-11 | *FADS2* |
| UNK30 | LPC (20:4)_rp_a  (Corr: 0.62) | | 11:61609750 | | C | T | 0.58 | -87.54 | 199.652 | 5.80E-10 | *FADS2* |
| UNK31 | Eicosatetraenoic acid  (Corr: 0.51) | | 11:61593816 | | C | T | 0.57 | -112.25 | 209.746 | 9.15E-15 | *FADS2* |
|  |  |  | 11:61640379 | | A | G | 0.58 | -96.12 | 242.243 | 6.58E-10 | *-* |
| UNK32 | None | | 14:67975822 | | C | A | 0.54 | 168.49 | 244.628 | 4.65E-27 | *TMEM229B* |
|  |  | | 14:67987543 | | C | T | 0.59 | 137.69 | 230.648 | 1.23E-19 | *-* |

1. Correlation coefficient between named metabolites and unnamed metabolites were calculated using Pearson correlation method
2. Abbreviation: Corr.:Correlation ceefficient; EPA: Eicosapentaenoic acid; LPC lysophosphatidylcholine; LPE lysophosphatidylethanolamine; MG: Monoacylglycerol; rp_a & rp_b: marking for the isomers

Supplementary table 2. Named metabolites list associated with glycemic traits, lipid traits, and nutrient intake

| Any glycemic traits: Fasting glucose, 2-hour post-load glucose, HbA1C, log-transformed insulin, log-transformed HOMA-IR | Any lipid traits: Cholesterol, HDL, Triglycerides,calculated LDL, non-HDL | Any nutrient intake: Carbohydrates, Protein, Total fat, saturated fat, monounsaturated fat, polyunsaturated fat |
| --- | --- | --- |
| Choline | LPC 20:4_rp_b | DG 32:1 |
| Ƴ-Glutamylisoleucine | DG 32:0 | Aminobutyric acid |
| Arachidic acid | DG 18:1_18:1 | PC 32:1 |
| Citramalic acid | LPC 20:3_rp_b | DG 18:1_18:1 |
| DG 32:1 | DG 34:1 | CAR 5:1 |
| Docosapentaenoic acid | Ketoleucine | 3-Hydroxybutyric acid |
| Docosatrienoic acid | SM d32:1 | DG 32:0 |
| Docosenoic acid | PC 34:2 | DG 34:2 |
| Dodecenoic acid | MG 18:1 | DG 34:1 |
| Eicosadienoic acid | LPC 20:3_rp_a |  |
| Eicosatetraenoic acid | PC 32:0 |  |
| Eicosatrienoic acid | PC 34:4 |  |
| Eicosenoic acid | SM d32:2 |  |
| EPA | SM d35:1 |  |
| Hydroxydodecanoic acid | CAR 18:0 |  |
| Hydroxyhexadecanoic acid | PC 28:0 |  |
| Hydroxyphenyllactic acid | DG 36:3 |  |
| Margaric acid | 3-Methyl-2-oxovaleric acid |  |
| MG 14:0 | DG 34:2 |  |
| Myristoleic acid | SM d38:1 |  |
| N-Acetylneuraminic acid | 3-Hydroxybutyric acid |  |
| Octadecadienoic acid | SM d36:1 |  |
| Octadecatrienoic acid | DG 32:1 |  |
| Oleic acid | LPC 14:0 |  |
| Palmitic acid | CAR 20:0 |  |
| Palmitoleic acid | LPC 15:0_rp_a |  |
| PC 32:1 | LPC 16:0_rp_a |  |
| PC 34:4 | PC 32:2 |  |
| PC 35:3 | LPE 18:0_rp_a |  |
| Stearic acid | LPC 20:4_rp_a |  |
| 2-Hydroxybutyric acid | LPC 16:1 |  |
| Ƴ-Glutamylleucine | Leucine |  |
| DHA | CAR 5:1 |  |
| Docosatetraenoic acid | Deoxyguanosine |  |
| Heptadecanedioic acid | Cortisol |  |
| Hydroxytetradecanoic acid | Uridine |  |
| Nonadecenoic acid | SM d42:2 |  |
| 2-Deoxyglucose | Ƴ-Glutamylisoleucine |  |
| 3-Hydroxybutyric acid | Isoleucine |  |
| 3-Methyl-2-oxovaleric acid | α-Tocopherol |  |
| DG 18:1_18:1 | Ƴ-Glutamylleucine |  |
| DG 32:0 | Cytidine |  |
| DG 34:1 | Leucine Isoleucine |  |
| DG 34:2 | MG 14:0 |  |
| DG 36:3 | MG 16:0_rp_a |  |
| Glutamine | PC 32:1 |  |
| Isoleucine | Leucine and isoleucine combined quantity |  |
| Ketoleucine | PC 34:3 |  |
| Leucine | SM d40:2 |  |
| Leucine and isoleucine combined quantity | Cholesterol -H2O |  |
| MG 16:0_rp_a | Citramalic acid |  |
| Leucine Isoleucine | Cerotic acid |  |
| N-Acetylglycine | PC 30:0 |  |
| Ƴ-Glutamyltyrosine | PC 36:3 |  |
| 3-Methylbutyrylcarnitine | Pipecolic acid |  |
| Phenylalanine | 3-Methylbutyrylcarnitine |  |
| Glu-Phe | SM d36:2 |  |
| CAR 4:0 | Hydroxyphenyllactic acid |  |
| CAR 5:0 isomers | CAR 5:0 isomers |  |
| CAR 3:0 | SM d36:3 |  |
| CAR 5:0 | CAR 12:0 |  |
| Adipic acid | Eicosatetraenoic acid |  |
| Tryptophan | CAR 14:0 |  |
| Kynurenine | CAR 10:0 |  |
| N-Acetylleucine | SMd35:1 |  |
| Taurochenodeoxycholic acid | Eicosatrienoic acid |  |
| Taurocholic acid | Cortisone |  |
| MG 18:1 | Indolelactic acid |  |
| Glycocholic acid-Glycohyocholic acid | Docosatetraenoic acid |  |
| Pro-Phe | CAR 6:0 |  |
| Tyrosine |  |  |
| Caffeine |  |  |
| Behenic acid |  |  |
| Biliverdin |  |  |
| PC 40:6 |  |  |
| N-Acetylserine |  |  |
| PC 34:3 |  |  |
| Uric acid |  |  |
| PC 36:3 |  |  |
| Glycochenodeoxycholic acid |  |  |
| dUMP |  |  |
| PC 32:2 |  |  |
| N-Methyl-D-aspartic acid |  |  |
| Chenodeoxycholic acid-Deoxycholic acid |  |  |
| Glycodeoxycholic acid |  |  |

Supplementary table 3. Unnamed metabolites with significant SNP-nutrient interactions

| Unnamed Metabolites | Most correlated named metabolites | | Nutrient | SNP | | Effect  Allele | | AF | β  G-nut. | P-Value  Interaction | P-Value  Joint | GMMAT  p-value |
| --- | --- | --- | --- | --- | --- | --- | --- | --- | --- | --- | --- | --- |
| UNK33 | Biliverdin  (Corr: 0.87) | Carb. | | | 5:30599586 | | G | 0.21 | 0.048 | 1.84E-08 | 2.08E-08 | 0.055 |
|  |  | Carb. | | | 5:30600589 | | A | 0.22 | 0.048 | 1.65E-08 | 1.79E-08 | 0.052 |
|  |  | Carb. | | | 5:30602495 | | C | 0.22 | 0.048 | 1.65E-08 | 1.79E-08 | 0.052 |
|  |  | Carb. | | | 5:30602910 | | T | 0.22 | 0.048 | 1.65E-08 | 1.79E-08 | 0.052 |
|  |  | Carb. | | | 5:30604771 | | C | 0.22 | 0.048 | 1.90E-08 | 1.97E-08 | 0.050 |
|  |  | Carb. | | | 5:30607792 | | C | 0.22 | 0.048 | 1.90E-08 | 1.97E-08 | 0.050 |
|  |  | Carb. | | | 5:30608507 | | T | 0.22 | 0.048 | 1.90E-08 | 1.97E-08 | 0.050 |
|  |  | Carb. | | | 5:30611219 | | TA | 0.22 | 0.048 | 1.90E-08 | 1.97E-08 | 0.050 |
|  |  | Carb. | | | 5:30611639 | | A | 0.22 | 0.048 | 1.90E-08 | 1.97E-08 | 0.050 |
|  |  | Carb. | | | 5:30612899 | | T | 0.22 | 0.048 | 1.90E-08 | 1.97E-08 | 0.050 |
|  |  | Carb. | | | 5:30613836 | | A | 0.22 | 0.048 | 1.90E-08 | 1.97E-08 | 0.050 |
|  |  | Carb. | | | 5:30615569 | | T | 0.22 | 0.048 | 1.90E-08 | 1.97E-08 | 0.050 |
|  |  | Carb. | | | 5:30617557 | | C | 0.22 | 0.048 | 1.90E-08 | 1.97E-08 | 0.050 |
|  |  | Carb. | | | 5:30617938 | | T | 0.22 | 0.048 | 1.90E-08 | 1.97E-08 | 0.050 |
|  |  | Carb. | | | 5:30623762 | | C | 0.78 | -0.048 | 1.90E-08 | 1.97E-08 | 0.050 |
|  |  | Carb. | | | 5:30628417 | | T | 0.78 | -0.048 | 1.90E-08 | 1.97E-08 | 0.050 |
|  |  | Carb. | | | 5:30629256 | | T | 0.78 | -0.048 | 1.90E-08 | 1.97E-08 | 0.050 |
|  |  | Carb. | | | 5:30629646 | | A | 0.78 | -0.048 | 1.90E-08 | 1.97E-08 | 0.050 |
|  |  | Carb. | | | 5:30630563 | | T | 0.78 | -0.048 | 1.90E-08 | 1.97E-08 | 0.050 |
|  |  | Carb. | | | 5:30634884 | | G | 0.78 | -0.048 | 1.90E-08 | 1.97E-08 | 0.050 |
|  |  | Carb. | | | 5:30635164 | | G | 0.78 | -0.048 | 1.90E-08 | 1.97E-08 | 0.050 |
|  |  | Carb. | | | 5:30641038 | | T | 0.78 | -0.048 | 1.90E-08 | 1.97E-08 | 0.050 |
|  |  | Carb. | | | 5:30641512 | | C | 0.78 | -0.048 | 1.90E-08 | 1.97E-08 | 0.050 |
|  |  | Carb. | | | 5:30645124 | | T | 0.78 | -0.048 | 2.03E-08 | 1.98E-08 | 0.046 |
|  |  | Carb. | | | 5:30649097 | | A | 0.78 | -0.048 | 1.90E-08 | 1.97E-08 | 0.050 |
| UNK34 | None | Protein | | | 3:6249007 | | T | 0.10 | 0.158 | 2.17E-08 | 1.38E-08 | 0.030 |
| UNK35 | None | Sat. Fat | | | 11:103681759 | | T | 0.04 | 0.370 | 3.32E-08 | 3.09E-08 | 0.048 |
|  |  | Sat. Fat | | | 11:103683709 | | G | 0.04 | 0.370 | 3.32E-08 | 3.09E-08 | 0.048 |
|  |  | Sat. Fat | | | 11:103689442 | | A | 0.04 | 0.364 | 4.53E-08 | 4.11E-08 | 0.046 |
|  |  | Sat. Fat | | | 11:103693019 | | G | 0.04 | 0.364 | 4.53E-08 | 4.11E-08 | 0.046 |
|  |  | Sat. Fat | | | 11:103694149 | | A | 0.04 | 0.370 | 3.32E-08 | 3.09E-08 | 0.048 |
|  |  | Sat. Fat | | | 11:103700803 | | A | 0.04 | 0.370 | 3.32E-08 | 3.09E-08 | 0.048 |
|  |  | Sat. Fat | | | 11:103702022 | | C | 0.04 | 0.364 | 4.53E-08 | 4.11E-08 | 0.046 |
|  |  | Sat. Fat | | | 11:103702225 | | C | 0.04 | 0.370 | 3.32E-08 | 3.09E-08 | 0.048 |
|  |  | Sat. Fat | | | 11:103710896 | | T | 0.04 | 0.364 | 4.53E-08 | 4.11E-08 | 0.046 |
|  |  | Sat. Fat | | | 11:103714138 | | C | 0.04 | 0.370 | 3.32E-08 | 3.09E-08 | 0.048 |
|  |  | Sat. Fat | | | 11:103715726 | | C | 0.04 | 0.364 | 4.53E-08 | 4.11E-08 | 0.046 |
|  |  | Sat. Fat | | | 11:103717473 | | T | 0.04 | 0.370 | 3.32E-08 | 3.09E-08 | 0.048 |
| UNK1 | Octadecadi-enoic acid  (Corr 0.74) | Sat. Fat | | | 20:40382240 | | C | 0.51 | 0.147 | 3.84E-08 | 4.71E-08 | 0.063 |
| UNK36 | SM(d36:3)  (Corr:0.54) | Total Fat | | | 1:187747584 | | T | 0.27 | -0.063 | 3.69E-09 | 2.63E-08 | 0.638 |
|  |  | Mon. Fat | | |  |  |  |  | -0.144 | 3.05E-09 | 2.11E-08 |  |
|  |  | Total Fat | | | 1:187737758 | | T | 0.28 | -0.062 | 9.48E-09 | 6.93E-08 | 0.806 |
|  |  | Mon. Fat | | |  |  |  |  | -0.133 | 3.83E-08 | 2.66E-07 |  |
|  |  | Total Fat | | | 1:187737754 | | G | 0.28 | -0.060 | 1.66E-08 | 1.19E-07 | 0.782 |
|  |  | Mon. Fat | | |  |  |  |  | -0.130 | 3.91E-08 | 2.69E-07 |  |
|  |  | Total Fat | | | 1:187689364 | | C | 0.24 | -0.060 | 3.51E-08 | 2.19E-07 | 0.549 |
|  |  | Mon. Fat | | |  |  |  |  | -0.132 | 4.62E-08 | 2.76E-07 |  |
|  |  | Total Fat | | | 1:187689404 | | A | 0.24 | -0.060 | 3.51E-08 | 2.19E-07 | 0.549 |
|  |  | Mon. Fat | | |  |  |  |  | -0.132 | 4.62E-08 | 2.76E-07 |  |
|  |  | Total Fat | | | 1:187692534 | | A | 0.24 | -0.060 | 3.51E-08 | 2.19E-07 | 0.549 |
|  |  | Mon. Fat | | |  |  |  |  | -0.132 | 4.62E-08 | 2.76E-07 |  |
|  |  | Total Fat | | | 1:187693050 | | A | 0.24 | -0.060 | 3.51E-08 | 2.19E-07 | 0.549 |
|  |  | Mon. Fat | | |  |  |  |  | -0.132 | 4.62E-08 | 2.76E-07 |  |
|  |  | Total Fat | | | 1:187695514 | | G | 0.24 | -0.060 | 3.51E-08 | 2.19E-07 | 0.549 |
|  |  | Mon. Fat | | |  |  |  |  | -0.132 | 4.62E-08 | 2.76E-07 |  |
|  |  | Total Fat | | | 1:187698135 | | T | 0.24 | -0.060 | 3.51E-08 | 2.19E-07 | 0.549 |
|  |  | Mon. Fat | | |  |  |  |  | -0.132 | 4.62E-08 | 2.76E-07 |  |
|  |  | Total Fat | | | 1:187699089 | | A | 0.24 | -0.060 | 3.51E-08 | 2.19E-07 | 0.549 |
|  |  | Mon. Fat | | |  |  |  |  | -0.132 | 4.62E-08 | 2.76E-07 |  |
|  |  | Total Fat | | | 1:187699094 | | A | 0.24 | -0.060 | 3.51E-08 | 2.19E-07 | 0.549 |
|  |  | Mon. Fat | | |  |  |  |  | -0.132 | 4.62E-08 | 2.76E-07 |  |
|  |  | Total Fat | | | 1:187699305 | | C | 0.24 | -0.060 | 3.51E-08 | 2.19E-07 | 0.549 |
|  |  | Mon. Fat | | |  |  |  |  | -0.132 | 4.62E-08 | 2.76E-07 |  |
|  |  | Total Fat | | | 1:187701595 | | C | 0.24 | -0.060 | 3.51E-08 | 2.19E-07 | 0.549 |
|  |  | Mon. Fat | | |  |  |  |  | -0.132 | 4.62E-08 | 2.76E-07 |  |
| UNK37 | Tryptophan(Corr:0.83) | Total Fat | | | 1:14463067 | | A | 0.77 | 0.060 | 4.21E-08 | 2.85E-07 | 0.808 |
|  |  | Mon. Fat | | |  |  |  |  | 0.136 | 1.15E-08 | 8.20E-08 |  |

1. Correlation coefficient between named metabolites and unnamed metabolites were calculated using Pearson correlation method
2. Abbreviation: Carb: Carbohydrate; Corr.: Correlation ceefficient; Sat.Fat: Saturated Fat; Mon.Fat: Monounsaturated Fat; SM: Sphingomyelin
